# Quality control practices in FMRI analysis: Philosophy, methods and examples using AFNI

**DOI:** 10.3389/fnins.2022.1073800

**Published:** 2023-01-30

**Authors:** Richard C. Reynolds, Paul A. Taylor, Daniel R. Glen

**Affiliations:** Scientific and Statistical Computing Core, NIMH, NIH, Bethesda, MD, United States

**Keywords:** FMRI, quality control, AFNI, resting state, reproducibility, processing, data visualization, task-based

## Abstract

Quality control (QC) is a necessary, but often an under-appreciated, part of FMRI processing. Here we describe procedures for performing QC on acquired or publicly available FMRI datasets using the widely used AFNI software package. This work is part of the Research Topic, “Demonstrating Quality Control (QC) Procedures in fMRI.” We used a sequential, hierarchical approach that contained the following major stages: (1) GTKYD (getting to know your data, esp. its basic acquisition properties), (2) APQUANT (examining quantifiable measures, with thresholds), (3) APQUAL (viewing qualitative images, graphs, and other information in systematic HTML reports) and (4) GUI (checking features interactively with a graphical user interface); and for task data, and (5) STIM (checking stimulus event timing statistics). We describe how these are complementary and reinforce each other to help researchers stay close to their data. We processed and evaluated the provided, publicly available resting state data collections (7 groups, 139 total subjects) and task-based data collection (1 group, 30 subjects). As specified within the Topic guidelines, each subject’s dataset was placed into one of three categories: Include, exclude or uncertain. The main focus of this paper, however, is the detailed description of QC procedures: How to understand the contents of an FMRI dataset, to check its contents for appropriateness, to verify processing steps, and to examine potential quality issues. Scripts for the processing and analysis are freely available.

## Introduction

Quality control (QC) is a vital part of FMRI analyses, although it is often not detailed in studies or presentations. The presence of poor quality data can reduce the power and generalizability of results. Undetected non-physiological artifacts can greatly skew outcomes and alter study results. Importantly, some exclusionary criteria could also systematically bias results away from an accurate interpretation of the data and underlying brain behavior.

In theory, FMRI QC appears to be a straightforward process: Sort a data collection into “good” datasets to use, and “bad” datasets to exclude. Some set of metrics or quantities can be calculated to do this screening automatically, and then processing can proceed with the good subset. In practice, however, QC is a notably more challenging procedure because of the combined complexities and varieties of both FMRI acquisition and analyses.

We consider QC to be an integral part of the processing itself, rather than a separate step, because what it means to be a “usable” dataset depends on the processing steps and design of the final analysis. Consider a few basic examples:

1. The cerebellum in a subject’s dataset is truncated by the acquisition field of view (FOV): This subject’s data might be included in the final analysis of a purely cortical study but excluded in the case of a cerebellar-specific or whole brain study.
2. EPI signal strength and distortions can vary across the brain. Having a low temporal signal-to-noise ratio (TSNR) within the basal forebrain region might exclude a subject from a subcortical study, but not from one of the visual cortex.
3. Subject motion is one of the most difficult effects to account for within any study, particularly in resting state FMRI where it can drastically influence results. How many time points can be censored before a subject is deemed to have “too much” motion to include, and does this number change if one is studying a group that is predisposed to motion (e.g., young adolescents or Parkinson’s disease patients)? And what is even the “correct” censoring limit to utilize?

In this paper we describe a number of QC measures for both task-based and non-task (e.g., resting state or naturalistic) FMRI processing that are implemented in the AFNI software suite (Cox, 1996). This paper is part of a community-wide FMRI open QC project, “Demonstrating Quality Control (QC) Procedures in fMRI,” where various groups of developers and researchers detail their own methods for QC of data. Specifically, we note the following goals and procedures from the Project description:^1^

*This project aims to showcase examples of QC practices across institutions and to foster discussions within the field. Here, we welcome researchers and developers across the globe to describe their QC methods in detail and to show them “in action” for a varied dataset acquired across multiple sites and scanners*… *We welcome researchers to present their quality control assessments of the subjects in the provided data collection, listing which would be included or excluded from further analyses, and which might be considered borderline or “uncertain.”*

Our own perspective is based on our individual and collective experiences as researchers, collaborators, educators and software developers of the AFNI toolbox. The design principle of the AFNI toolbox is, “To help keep researchers close to their data,” and this influences our view of QC measures, as well. Rather than viewing QC as simply filtering datasets into “good” or “bad” bins, we regard it as the larger procedure of *being as sure as possible about the contents of the data collection, from acquisition properties to artifact checking to regression evaluation.* We note that some QC steps are quantitative (they can be derived directly from one or more numbers), some are qualitative (e.g., they require visualization) or a combination. Some involve interactively investigating the datasets in a GUI, which can be facilitated in AFNI by scripting. Some QC items can be evaluated “per subject” and are essentially independent of any other member of the data collection, while others involve the relative comparison of a property.

Here, we detail a set of QC procedures for FMRI subjects and provide examples of applying these to the Project datasets. The first stage of QC can occur before any real “processing” of datasets has taken place, called “getting to know your data” (GTKYD). It is not necessarily part of inclusion/exclusion criteria, but it importantly ensures consistency of acquisition parameters and data properties. Next, systematic quantitative and qualitative stages are set up directly within afni_proc.py’s processing pipeline and QC HTML: APQUANT and APQUAL, respectively. For task-based FMRI, the STIM stage investigates the stimulus event and timing information. Finally, the GUI (graphical user interface) stage should always be used for some set of subjects in a study, to verify dataset properties in depth, and it can also be useful for investigating unknown features that may be found in other QC stages. In short, we implement a wide variety of QC procedures to be detailed, and we partition these into conceptual groupings in order to aid systematization. We aim to be as descriptive as possible, to provide a starter guide for possible QC during FMRI processing.

## Methods: Data and processing

The datasets downloaded from the Project website and analyzed here were originally distributed as part of the following public repositories, according to the Project instructions: Functional Connectome Project (FCP; Biswal et al., 2010), ABIDE (Di Martino et al., 2014), and OpenNeuro (Markiewicz et al., 2021). They are due to be specifically identified in detail in a future publication of the Project, but we note that each subject’s dataset was acquired in a single session at 3T using a single echo EPI sequence, and overall they have fairly “typical” acquisition parameters (in terms of TR, voxel size, etc.—see below).

Here, AFNI v23.3.02 (Cox, 1996) and FreeSurfer v7 (Fischl and Dale, 2000) software packages were used for processing each of the resting state and task-based FMRI data collections. For each collection, AFNI’s afni_proc.py was used to set up the full FMRI processing pipeline, which runs through regression modeling and includes an automatically generated quality control (APQC) HTML report. The full set of scripts in each case are available online: https://github.com/afni/apaper_afniqc_frontiers.

As noted in the Introduction, some QC details rely on processing choices and on the analysis being performed. In the present Project, there was no stated group analysis, so we considered investigating these datasets in preparation for a generic cortical, voxelwise analysis. For this QC, we note issues regarding issues in cerebellum or midbrain, but do not exclude subjects based on these (these regions were often excluded or only partially included in the EPI FOVs).

### Resting state FMRI data and processing

The provided resting state data collection consists of acquisitions from seven different sites, each of approximately 20 subjects, with a total of 139 subjects. Each site is signified by the hundreds-digit of the subject ID, by which we refer to each subset. That is, Group 1 contains sub-101, sub-102, etc.; Group 2 contains sub-201, sub-202, etc.

For each subject, there is one T1w anatomical and one EPI time series, except within Group 6, in which several (but not all) subjects have two EPI time series. The whole brain, T1w anatomical volumes typically have voxels with approximately 1.0 mm resolution, though there is some inter- and intra-group heterogeneity. The EPI time series have the following ranges of properties: TR = 2.0–2.5 s; minimum voxel edge = 1.56–4.00 mm, and maximum voxel edge = 3.10–4.00 mm (with varied anisotropy); in-plane matrix size = 64–128, and through-plane matrix size = 32–47; number of volumes (per run) = 123–724. Four out of seven sites had acquired (at least some) EPI and anatomical volumes obliquely. Further details about the heterogeneity of basic dataset properties are enumerated within the first stage of QC results (GTKYD), below.

The first step of processing was to run FreeSurfer’s recon-all on each T1w anatomical volume, providing an initial brain mask and parcellations for reference. FreeSurfer parcellations were entered into afni_proc.py as “follower” datasets, to be mapped to the final template space and to provide optional reference locations there. Note that if performing an ROI-based analysis, blurring would typically not be included in the processing steps. AFNI’s @SSwarper program was also run on each T1w volume, to provide both a final skullstripping (SS) mask and a non-linear warp [*via* 3dQwarp; Cox and Glen (2013)] from that anatomical to the MNI-2009c (asymmetric) template space [Fonov et al. (2011)]. Identical @SSwarper commands were used for Groups 1–6, and for Group 7 a different cost function (nmi, normalized mutual information, instead of lpa, local pearson correlation absolute value) was utilized to improve results. These outputs of @SSwarper were included in the afni_proc.py command, described below.

AFNI’s afni_proc.py program was used to generate a full, reproducible FMRI processing pipeline across each Group. While the afni_proc.py command contains the specified “control variables” of each processing block, the created script (which is automatically commented) can also be read to understand the exact implementation details. Because each resting state group was acquired with slightly different parameters, particularly voxel size, individual afni_proc.py commands were created here for each so that parameters such as “applied blur” would be appropriate for each. In an expressly multisite study, which would combine subjects across all sites/Groups into a single analysis, this approach might differ—for example, one might apply an option to blur all EPI datasets to the same full-width at half-max (FWHM) value, for final uniformity. Here, the only parameters that varied across each group’s afni_proc.py commands were the values of the applied blur size (“-blur_size”) and final EPI voxel dimensions (“-final_dxyz”).

The afni_proc.py processing included initial despiking and slice timing correction. The EPI volume with the minimum fraction of outliers in the brain mask was selected to be a reference for motion correction (rigid-body alignment across the FMRI time series) and EPI-anatomical alignment (linear affine transformation with 12 degrees of freedom). EPI-anatomical alignment was calculated by first creating a brightness-homogenized version of the reference EPI volume and then using the “lpc+ZZ” cost function for local Pearson correlation (Saad et al., 2009). For anatomical-template alignment, the non-linear warp from the previous @SSwarper step was included. An EPI volume extents mask was applied to omit voxels that, due to motion, did not have acquired data throughout the entire time course. An EPI brain coverage mask was generated for the purpose of calculating statistics, but following the default behavior in afni_proc.py, this mask was not otherwise applied, leaving the time series basically unmasked, allowing for more complete QC (we recommend masking at the group level). A Gaussian blur was applied to each time series, with FWHM of approx. 1.5–2x the mean EPI voxel dimension (see scripts). Time series were scaled to have a mean of 100, to put the data in units of percent change. This scaling has a negligible effect on correlations, though it is helpful if computing parameters such as fALFF, for example.

The final processing block within the afni_proc.py command includes regression modeling, which amounts to projection of signals of non-interest, in the case of resting state analysis. This included censoring, for volumes with Enorm (Euclidean norm of first differences of motion parameters) >0.2 mm or an outlier fraction >5% within a whole brain mask. Default polynomial regressors were used to model the slow baseline drifts. The six time series from rigid-body EPI alignment and each of their derivatives were included “per-run” as motion regressors. Bandpassing within the standard low frequency fluctuation (LFF) range of approx. 0.01–0.1 Hz was *not* included in this processing, since it has been shown that useful physiological data exist in the FMRI time series above 0.1 Hz (e.g., Gohel and Biswal, 2015; Shirer et al., 2015), and such bandpassing incurs a large statistical cost in terms of degrees of freedom (Caballero-Gaudes and Reynolds, 2017). The consequences for FMRI QC of including standard LFF range bandpassing are discussed below.

### Task-based FMRI data and processing

The provided task-based data collection consists of 30 subjects (subject IDs: sub-001, sub-002, etc.) acquired at a single site. A single task paradigm was used, and timing files were provided in both original BIDS format and in a simplified, columnar format. For each subject, there is one T1w anatomical and one EPI time series. The whole brain, T1w anatomical volumes have 1.00 mm isotropic voxels. The EPI time series have the following properties: TR = 2.0 s; voxel dimensions = 3.00 mm × 3.00 mm × 4.00 mm; matrix dimensions = 64 × 64 × 34; number of volumes = 242; oblique slices.

As for the resting state processing above, FreeSurfer recon-all and AFNI @SSwarper commands were run on each subject’s T1w anatomical volumes. In setting up stimulus timing, we note that there are many ways to interpret and make a model from the event files. We chose to model the 2 event types, Task and Control, using reaction time for event duration, and the full duration if a subject did not respond in time. Control events had durations between 0 and 2 s, while task events lasted between 0 and 4 s. AFNI’s timing_tool.py was used to apply this interpretation.

In the task-based afni_proc.py, the same processing blocks and options for slice timing correction, intra-EPI registration (for motion correction), EPI-anatomical alignment, anatomical-template alignment, mask estimation and scaling. The despiking block was not used. The blur size was set to 6 mm, the application of which was restricted to the estimated mask.

The regression model included censoring for volumes with Enorm ≥0.3 mm (a slightly higher value than for the resting state processing, since the latter tends to be more sensitive to motion effects) or an outlier fraction >5% within a whole brain mask. The six time series from rigid-body EPI alignment were included per-run as motion regressors. In the task design, there were two stimulus classes: “Task” and “Control” events (the latter name should not be confused with the standard subject specification of “control group”; also, in this Project, there were no such group classifications). These were modeled as duration modulated blocks, normalized to a 2 s response time [“-regress_basis_multi ‘dmUBLOCK(-2)”’], and serial correlation within the time series was accounted for with 3dREMLfit (“-regress_reml_exec”). Two general linear tests (GLTs) were specified as potential conditions of interest: The contrast “Task - Control,” and the average stimulus response “0.5* (Task + Control).”

### General, simple and fast FMRI “quick” processing

The previous two sections describe the detailed processing options selected for the resting state and task-based processing commands implemented for these specific data collections. For each, several processing options and control parameters are selected by the user, tailored to the study design and research question. These are useful and appropriate for full dataset processing, e.g., as part of a group analysis.

However, we note an additional tool called ap_run_simple_rest.tcsh that is much simpler to set up for quick, general processing for any FMRI dataset; it is particularly useful for QC purposes. The AFNI program is a wrapper for afni_proc.py with a particularly simple front end: The only required options are the input dataset names (some additional ones can be entered, too). Importantly, this program can be used to generate the vast majority of the QC information that is detailed below. In particular, almost every quantitative QC criterion (described under APQUANT) should be essentially identical.

This alternative analysis tool was designed with the focus of providing efficient checks for datasets as individual subjects are acquired, and can even be implemented to perform QC while the subject is in the scanner—thus, data could be reacquired if there were a particular problem such as severe motion or EPI dropout. Similarly, it could be easily created automatically as the scanner saves data to storage, to generate a uniform QC HTML report that would be immediately available to all researchers acquiring data. This tool uses affine template registration and processes data as resting state, making it simple, fast, and suitable to provide detailed QC. While the seed-based correlation QC maps can be considered slightly noisier than in a full processing case that implements non-linear alignment, they should still be reasonable and useful for quick QC purposes. In this work, we describe the QC items using the specific afni_proc.py commands, but the same considerations would apply to the “quick” outputs here.

## Procedures for FMRI quality control

**FIGURE 1:**
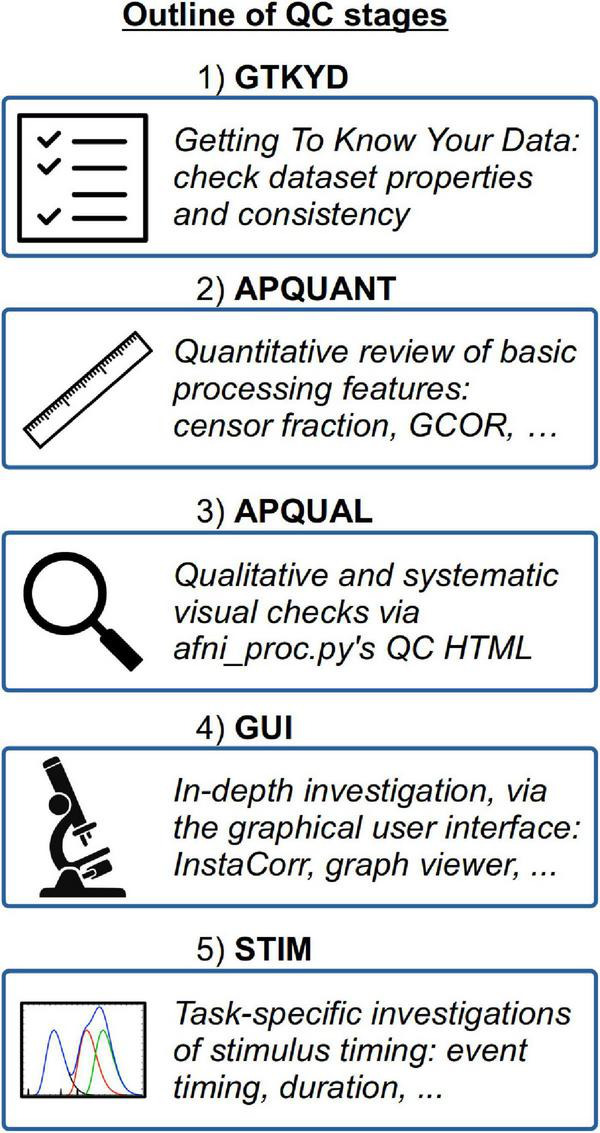
An overview of the QC stages presented in the current study.

A schematic overview of the QC stages is shown in Figure 1.

### 1) GTKYD: Getting to know your data

The first stage in the QC procedure here is referred to as GTKYD, which has two primary features. First, this checks the consistency of several key data and header properties within the group, such as dataset orientation, matrix size and more. Second, this investigates the reasonableness of the dataset properties, such as voxel size (units and isotropy), minimum/maximum values within the volumes (for possible scanner saturation) and more. Problematic values in either of these “relative” and “absolute” checks, respectively, might be a sign of acquisition mistake, DICOM-to-NIFTI conversion trouble, incorrect header information, BIDS construction, or other errors when creating the collection.

In general, this GTKYD stage is not intended to be used to include/exclude individual subjects. Instead, its purpose is to verify that the datasets contain their expected properties and are appropriate for the analysis at hand. Questions or potential issues should lead to double-checking the acquisition sequence and reconstruction steps, whether collected by the researchers performing the analysis, or, for public or shared data, by contacting those who did acquire it. In the first case, we recommend performing the QC steps immediately and repeatedly as each subject in a study is collected, to protect against long-running and fundamental issues in the data, which may lead to wasted acquisition time and expense. In all cases, GTKYD reduces the possibility of analyzing fundamentally problematic or inappropriate data.

The GTKYD properties checked here included the following for both EPI and anatomical volumes:

• header-info: Matrix size, orientation, voxel dimensions, datum type, NIFTI qform_code, NIFTI sform_code
• data-info: Number of runs, minimum value, maximum value.

Additionally, the following was checked for EPI:

• header-info: TR, number of time points, slice timing.

### 2) APQUANT: Quantitative review of basic processing features

This stage describes the automation of quantitative QC measures output during afni_proc.py processing. This includes scriptable subject exclusion criteria, as well as checks for processing consistency and additional warnings. The output of this stage, created by AFNI’s gen_ss_review_table.py (GSSRT) program, is a list of subjects to exclude/include.

During processing with afni_proc.py, a results directory is created for the full output, including storage of intermediate datasets, text files, and other information. In particular for this QC step, a single file of “basic” review quantities related to the processing is made. This essentially contains a dictionary of summary information about the processing—such as software version used, input datasets, censor fractions, and more—for each subject. For example, the “TRs censored” field records how many time points were censored during the subject’s processing, “motion limit” records the threshold value used for Enorm censoring, and “global correlation GCOR” records the average correlation across all pairs of brain-masked voxels. These single subject review dictionaries can be combined across the group into an information table, using GSSRT, with one subject per row and one dictionary key (or review field) per column.

Importantly, one can provide a “checklist” of features to query, and create a sub-table of subjects that have one or more properties. For example, one could apply a set of exclusion criteria by generating a subtable of all subjects who have too many censored time points or too low an average TSNR. Additionally, one can create descriptive tables to verify that all subjects had similar EPI voxel sizes and were analyzed with the same software version. This combination of afni_proc.py’s basic processing dictionary and GSSRT’s table-generating functionality is very flexible and useful for staying informed about a wide range of properties about the data processing as a whole.

Here, we created three separate review tables for each group: One for checking the analysis consistency across subjects; one for checking for possible concerns in the data at a “warning” level; and one for applying strict exclusion criteria. The GSSRT fields and comparison operators for each table’s checklist are shown in Table 1. As noted in the table, all the same criteria were applied to both resting state and task-based FMRI collections, with one additional exclusion criterion for the latter. Additional criteria could be selected, as well, depending on the study. For example, while it was not used in this study, a Dice coefficient for the overlap between the EPI mask and the anatomical mask would be useful for cases where a specified minimum fraction of brain coverage is required.

**TABLE 1:** Lists of QC criteria for generating review tables of different properties after completing single subject processing with afni_proc.py.

APQUANT checklists (rest and task FMRI)		
**Consistency checklist (rest, task)**		
Key/field	Comp.	Description

’AFNI version’	VARY	Does the package version vary?
’num regs of interest’	VARY	Does the number of regressors of interest vary?
’final voxel resolution’	VARY	Do the final voxel dimensions vary?
’num TRs per run’	VARY	Does the number of EPI time points per run vary?
**Warnings checklist (rest, task)**		
Key/field	Comp.	Description

’final DF fraction’	LE 0.7	Is the remaining fraction of degrees of freedom >= 0.7? (NB: Bandpassing would affect this.) Visualize DF summary in APQC ‘regr’ block.
’censor fraction’	GE 0.15	Is the fraction of censored time points >= 0.15?
’average censored motion’	GE 0.1	After censoring, is the remaining average motion (Enorm) >= 0.1 mm?
’max censored displacement’	GE 6	Are any two volumes >= 6 mm apart?
’global correlation (GCOR)’	GE 0.15	Is GCOR >= 0.15? Visualize in APQC ‘regr’ block as corr_brain.
’TSNR average’	LT 150	Is the within-mask average TSNR <= 150? Visualize in APQC ‘regr’ block as TSNR-final.
**Exclusion criteria checklist (rest)**		
Key/field	Comp.	Description

’final DF fraction’	LE 0.6	Is the remaining fraction of degrees of freedom <= 0.6? (NB: Bandpassing would affect this.) Visualize DF summary in APQC ‘regr’ block.
’censor fraction’	GE 0.2	Is the fraction of censored time points >= 0.2?
’average censored motion’	GE 0.15	After censoring, is the remaining average motion (Enorm) >= 0.15 mm?
’max censored displacement’	GE 8	Are any two volumes >= 8 mm apart?
’global correlation (GCOR)’	GE 0.20	Is GCOR >= 0.20? Visualize in APQC ‘regr’ block as corr_brain.
’flip guess’	EQ DO_FLIP	Is there an EPI-anatomical left-right flip? Visualize in APQC ‘warns’ block.
**Exclusion criteria checklist (task)**		
Key/field	Comp.	Description

’final DF fraction’	LE 0.6	Is the remaining fraction of degrees of freedom <= 0.6? (NB: Bandpassing would affect this.) Visualize DF summary in APQC ‘regr’ block.
’censor fraction’	GE 0.2	Is the fraction of censored time points >= 0.2?
’average censored motion’	GE 0.15	After censoring, is the remaining average motion (Enorm) >= 0.15 mm?
’max censored displacement’	GE 8	Are any two volumes >= 8 mm apart?
’global correlation (GCOR)’	GE 0.20	Is GCOR >= 0.20? Visualize in APQC ‘regr’ block as corr_brain.
’flip guess’	EQ DO_FLIP	Is there an EPI-anatomical left-right flip? Visualize in APQC ‘warns’ block.
’fraction TRs censored’	GE 0.2	Is the fraction of time censored from any stimulus response >= 0.2?

These key or field values are automatically placed in a text file within each subject’s results directory by afni_proc.py. Each set of key/fields and comparisons (Comps.) is then used within a gen_ss_review_table.py command to create a summary table. The following comparison operators are used here: VARY = “differs across subjects”; GE = “greater than or equal to”; LE = “less than or equal”; and EQ = “equal to.” For each group of subjects, a set of consistency, warning and drop criteria tables were made.

It is important to note that the specific threshold values we have used for the quantitative keys could differ across studies. For example, rodent datasets would have much smaller head size and voxels, and one might expect less motion if they were anesthetized. One might allow for different motion criteria in a study of motion-prone children. The appropriateness of a particular TSNR threshold may vary with scanner. Over time, more knowledge may be accrued to inform better parameter selections, from the point of view of sensitivity and specificity. The present values seemed reasonable for this study and may form a starting basis for other ones, but should not be taken as absolute.

### 3) APQUAL: Qualitative and visual checks using afni_proc.py’s QC HTML

In complement to the APQUANT stage, this section describes performing a qualitative, visual-based assessment of the processing results. In particular, this is done using afni_proc.py’s QC report (APQC), which is an automatically generated HTML document. It is an interactive HTML for investigating various features of the data, including the original data, alignment, statistical maps and modeling, motion, warnings, and more. Ratings and comments can be saved for each QC block.

While some features of processing can be assessed quantitatively, many others essentially require visualization. For example, image registration is driven by a quantitative cost function, but then separate assessment is needed to verify that tissue boundaries and sulcal and gyral patterns appear to be well-aligned. Furthermore, there are numerous potentially artifactual patterns that can appear in datasets; these can be most easily identified by the human eye, and either recognized directly or marked for requiring further exploration. In many cases, fully understanding a subject’s dataset and problems that may exist with it requires having a multifaceted appreciation for it, and the APQC HTML provides one form of this.

The APQC HTML is organized in successive “QC blocks,” whose elements are grouped by processing steps and conceptual relatedness. Most blocks are common to both task-based and non-task processing, though some features are distinct (as noted in the descriptions below). Additionally, some features depend on the details of processing—e.g., the anatomical-to-template alignment block only exists if one is registering the subject to a template space. In the following, we describe the current QC blocks and features for single-echo FMRI processing. For each block, we provide a list of elements or keys that describe specific features in a QC assessment, and these terms are used when evaluating the present data collection in the Results section. These keys may provide a generalizable categorization for QC reporting. They are also likely to grow in number over time.

#### vorig

Views of the original space EPI (specifically, the volume registration reference) and anatomical volumes, as well as their overlap.*

• EPI: FOV coverage, signal dropout, ghosting overlap, poor tissue contrast (esp. if alignment fails), spatial distortion (see better check in ve2a), inhomogeneity.
• anat: FOV coverage, ringing, poor tissue contrast (esp. if alignment fails), inhomogeneity, skull stripping (if previously applied).
• overlap: Initial EPI/anat overlap (informational, in case EPI-anatomical alignment fails).

#### ve2a

Views of the EPI-to-anatomical alignment results: Anatomical edges overlayed on the EPI.*

• global: Overall quality of alignment (e.g., from sulcal, gyral and ventricle patterns; note CSF can affect the appearance of the outer edge).
• local: Part of volume matching is poor (particularly around regions of interest), which can be due to FOV coverage, EPI signal dropout, distortion, other.

#### va2t

Views of the anatomical-to-template results: Template edges overlayed on the anatomical.*

• global: Overall quality of alignment (e.g., from sulcal, gyral and ventricle patterns).
• local: Part of volume matching is poor due to, e.g., FOV coverage, distortion, SS, regional mismatch, other.

*One of the va2t, ve2a or vorig QC blocks will contain a view of the final EPI mask overlayed on the final reference volume, determined by whether the final space is a template, the subject’s anatomical or the subject’s EPI, respectively.

• mask-overlap: Estimated coverage of usable FMRI signal (typically intersected with the subject anatomical).

#### vstat

*Views of relevant statistical modeling. For non-task FMRI, when a recognized template space is used, seed-based correlation maps of the default mode, visual and auditory networks are shown. For task-based FMRI, the views include the full F-stat of modeling, as well as coefficient* + *stat maps of stimuli and contrasts of interest.^2^*

• quality: Overall expected/recognizable network correlation (or task statistical) patterns observed, such as full spatial coverage (no missing regions); network specificity (no extra regions); reasonable magnitude; extra-cranial patterns.
• artifact: Ghosting; striping; strong slice-based patterns; large spatial patterns across/unconstrained by tissue type; notably non-physiological patterns.

We note that in these images, and in several others within the APQC HTML, the thresholds are applied transparently. That is, suprathreshold regions are shown opaque (or with maximum translucency) and outlined, and subthreshold values are displayed with increasing transparency as the magnitude of the value decreases. This reduces the sensitivity to choice of threshold, and allows focal regions to be highlighted (with opacity and outlining) while still showing information throughout the brain (Allen et al., 2012; Taylor et al., 2022). Moreover, brain masks are typically not applied, to show results throughout the full FOV, which helps to further identify any potential artifacts.

#### mot

*Motion-related information: Plots of Enorm, outlier fraction, and motion parameter time series (with any censoring information shown), and a grayplot of residuals. Provides a useful reference (censor- and motion-related quantities are primarily checked across the group using GSSRT).*

• enorm: Odd patterns; regular signals, which are likely not physiological (e.g., mechanically driven); many time points with just sub-threshold values, which might drive spurious correlation (might lead to re-processing); overall value range.
• outliers: (same items as “enorm,” above); evaluate for synchrony against enorm.
• volreg-pars: Similar properties to “enorm” above; note that these parameters are not directly thresholded for censoring.
• grayplot: Strong vertical patterns may suggest high residual correlation (primarily checked in “regr-corr_brain” visualization and quantified in “qsumm” with GCOR).

#### regr

*Regression modeling information: Degree of freedom (DF) summary; view of correlation map with whole brain average residual signal (checks brainwide similarity of residuals, such as for large breathing, and motion effects remaining); and TSNR maps (good scenario: Relatively consistent TSNR around brain regions of interest). For task datasets, plots of the individual regressors of interest, as well as their sum, are shown (with any censor bands, for reference).*

• task-ideal-sum: (NB: Strongly paradigm dependent) any problem with the sum of regressors; large gaps and/or spikes might generally be worth noting.
• task-ideal-stim: (NB: Strongly paradigm dependent) any problem with an individual regressor of interest; duplicated stim timing (scripting mistake); stimulus-correlated motion may be worth noting.
• df-count: Too few degrees of freedom in output results (often due to censor fraction and/or bandpassing); typically checked automatically with GSSRT.
• corr_brain-artifact: (similar to vstat-artifacts) ghosting, correlation/anticorrelation striping, strong slice-based patterns, large spatial patterns across/unconstrained by tissue type, notably non-physiological patterns.
• corr_brain-quality: Too high (also typically quantified *via* GCOR and checked with GSSRT) or too low.
• TSNR_volreg: Mainly informational, since this is calculated before regression modeling and noise regression (look for similar features as in TSNR_final-* items).
• TSNR_final-loss: Notable dropout/low signal in regions of interest (e.g., often low in frontal/temporal lobes and subcortical nuclei).
• TSNR_final-artifact: Non-physiological patterns of TSNR magnitude, particularly dropout (e.g., vertical bands/stripes).

#### radcor

*Radial correlation maps: The value of correlating each voxel with a Gaussian-weighted local average (FWHM* = *40 mm in human datasets). A typically good scenario is relatively high values approximately constrained to GM; motion effects often appear as high correlation/anticorrelation patterns around the edge of the brain, which are often reduced after volreg.*

• tcat-artifact: Mainly informational, since this is calculated for initial data with no motion correction (look for similar features as in radcor_volreg-* items).
• volreg-artifact: Patches of high radcor values spanning multiple tissue types (can be sign of coil artifact or other non-physiological effects); artifacts here often inspire investigations with InstaCorr, as referred to in Procedure 4.

#### warns

*List of warnings from various checks throughout processing, including for: Regression matrix correlations; high censor fractions; pre-steady state outliers; left-right flip between input EPI and anatomical. Several can be checked with GSSRT (with useful details here for verification).*

• regr_mat: High pairwise correlations in regression matrix (varied).
• gen-censor: High total/overall censoring fraction (typically checked with GSSRT).
• task-stim-censor: High censoring fraction for one or more particular stimuli (optionally checked with GSSRT).
• press: EPI data appear to have pre-steady state volumes at the start (*via* outlier check; though sometimes this is simply due to motion in first time points).
• flip: EPI-anatomical might have relative flip, as checked with cost function alignment and to-be verified with provided images (Glen et al., 2020).

#### qsumm

*Basic quantitative information of processing, such as AFNI software version, voxel sizes; motion limits and counts; TSNR; and more. Provides a quick reference (many of these quantities should be checked across the group using GSSRT).*

• anomalous: An unexpected value, such as final voxel resolution, software version number, etc.
• suprathresh: Unexpectedly or problematically large value (e.g., censor fraction, GCOR).
• subthresh: Unexpectedly or problematically small value (e.g., average TSNR, maximum F-stat).
• missing: Quantity not present, perhaps due to coding error (e.g., missing censor fraction, missing censor fraction per run).

### 4) GUI: In-depth investigation with the graphical user interface

This stage describes exploring one or more datasets interactively. While this may require more time to perform than some other steps, it provides the best means for understanding things like the detailed alignment of two volumes, the combined spatio-temporal aspects of EPI time series (with “InstaCorr,” described here), etc. To facilitate this process, afni_proc.py automatically generates multiple scripts to load particular datasets and visualization functionality in the AFNI GUI.

• align: Check alignment (or registration) features.
• graph: View the time series plots of one or more voxels.
• instacorr: Flag peculiar spatio-temporal patterns in the time series data.
• other: Any other feature(s) using the afni and/or suma GUIs, plugins, etc.

#### align

There are a large number of features in the AFNI toolbox and GUI to inspect the alignment or registration between two datasets (e.g., see Appendix A in the Supplementary material of Glen et al., 2020). The default method is to show one volume as a grayscale background (underlay) dataset, while the other is shown in color as the “overlay” dataset. There are several methods for viewing the datasets interactive in different ways, depending on the properties of the datasets (matching or differing tissue contrasts, blurriness, etc.), which can help to focus on various features. These include: Toggling the underlay/overlay datasets, adjusting underlay contrast/brightness, adjusting overlay opacity, viewing the underlay edges, using a horizontal or vertical “image comparison” slider bar, and using a slider to fractionally blend the datasets.

#### graph

The AFNI GUI includes an interactive and expandable graph window for displaying the time series of one or more voxels. Observing properties of the time series, even when no stimulus has been provided, can provide useful insight, particularly into possible artifacts or non-neuronal confounds. For example, subject motion effects can be observed as peaks and sudden shifts in the amplitudes across many voxels. Drift or shimming-related changes can also be noted. One can also load a reference time series (e.g., one with the ideal task response) and investigate patterns, similarity or possible features showing stimulus-correlated motion.

#### InstaCorr

The InstaCorr functionality (which stands for “Instant Correlation”) within the AFNI GUI is the prime tool for an in-depth investigation of a 4D EPI dataset. Briefly, InstaCorr allows one to freely explore spatio-temporal patterns within a dataset by clicking and dragging a seed location anywhere throughout the volume; the resulting seed-based correlation patterns update continually and instantaneously, so that one can quickly assess a full FOV (see, e.g., Jo et al., 2010; Song et al., 2017). This is particularly useful for exploring functional networks, potential scanner artifacts, and more. Processing features such as baseline regression, bandpassing, smoothing, masking and setting a seed radius can all be selected within the InstaCorr setup menu. The afni_proc.py processing now automatically creates a “run_instacorr*.tcsh” script to run InstaCorr on the regression model’s output; running the script automatically opens the AFNI GUI with InstaCorr setup on the residuals dataset.

Here, we used InstaCorr in conjunction with the APQUAL step, when one or more QC images showed a questionable pattern. For example, we could observe whether there were: Large, non-physiological patches of high correlation; slice-constrained artifacts; and more. Resting state FMRI analysis often depends on correlation patterns, making InstaCorr verification particularly important. In task-based FMRI, it can provide useful exploration of areas where responses are unexpectedly low.

### 5) STIM: Task-specific investigations of stimulus timing

This stage describes understanding and evaluating the stimulus event timing for a task-based analysis. This includes answering whether events are presented at consistent intervals or randomized, of consistent duration or variable, and based on the subjects or not, both for duration and possibly amplitude modulators in the regression model. It includes answering similar questions for inter-stimulus intervals (ISIs). And it includes evaluating the stability of the regression matrix, i.e., whether small noise fluctuations could have a noticeable effect on the results.

There are several tools within AFNI that can be helpful for investigating various stimulus related features across the group, such as summaries of timing, duration and interstimulus intervals. These can be particularly useful in understanding variations or potential issues in subject results. Such investigations are essential during an experiment design phase, before acquiring subject data, and are similarly important for understanding event timing in a study from an external group, or even in review. Detailed investigations can be done for just a few subjects, while statistical reviews of stimulus durations and interstimulus interval timing can be performed and then summarized across all subjects, while looking for peculiarities or outlier subjects.

Two items that are often computed after the regression matrices exist are regressor correlations and condition numbers. Negative pairwise correlations are often expected, particularly in cases with two or just a few stimulus classes. As a measure of predictability, this happens when one stimulus response is “on” and another stimulus response is generally “off,” or lower. Such a pair of regressors might have a modestly high, negative correlation that is considered acceptable. Condition numbers (of the full model and conceptual sub-models) help identify when a model is becoming mathematically unstable, often from a stimulus design mistake, or by having too little non-stimulus time.

• events: (for just a few subjects) visually review event timing across all classes together, including onsets times, durations, and offsets from previous events, along with any modulators.
• stim-stats: Show min/mean/max/stdev of stimulus durations, per class and subject.
• isi-stats: Show min/mean/max/stdev of interstimulus intervals, per subject.
• X-cormat: (done in APQUANT.warns section, above) look for large pairwise correlations among the regression matrix regressors.
• X-cond: Look for high condition numbers across subsets of the regression matrix, including the baseline, motion terms, regressors of interest, and combinations of these sets up to the full matrix.

## Results for resting state data collections

### GTKYD summary

GTKYD was the first stage of checking each group’s data. In the present study, no subjects were excluded because of this stage’s results, but they did inform some processing choices (and in other cases, they indeed might lead to a group not being included in a study). Table 2 shows a summary of basic dataset properties that were inconsistent across a group. For example, in Group 5 six out of 20 subjects have an anatomical volume with differing orientation. This may reflect acquisition or reconstruction inconsistency, but importantly it may hide an error in correctly assigning directionality within the volume. While most mistaken “flips” of directionality within a dataset can be quickly detected visually, this is not so for left-right flips; for humans, relative EPI-anatomical flips can typically be reliably detected (Glen et al., 2020), but this is not the case for animal datasets or when all datasets for a subject are flipped.

**TABLE 2:** Summary of the first stage of resting state FMRI QC: GTKYD (“getting to know your data”).

GTKYD: “Getting To Know Your Data” results (resting state FMRI)	
**Property**	**Description**

**Group 1: EPI**	
matrix size diff	sub-118 has 112×112×47, from group std 96×96×47
num vols diff	sub-114 and sub-115 have 128, from group std 256
vox dim diff	sub-118 has 2.29×2.29×3.0 mm^3^, from group std 2.67×2.67×3.0 mm^3^

**Group 1: anatomical**	
matrix size diff	sub-104, sub-109, sub-112 and sub-117 have 256×180×256, from
	group std 256×200×256

**Group 2: EPI**	
large max values	approx. 2-4×10^6^
oblique	

**Group 2: anatomical**	
vox dim diff	sub-203 has 1×0.93×0.93 mm**3 from group std 1×1×1 mm**3
matrix size diff	sub-118 has 160×288×288, from group std 160×256×256

**Group 3:**	
no warnings	

**Group 4: EPI**	
no slice timing	

**Group 5: EPI**	
matrix size diff	sub-501, sub-502, sub-503, sub-504, sub-509 have 128×128×34,
	instead of group norm 80×80×35; others have 80×80×35 and 80×80×39
	mm^3^ instead of group std 3.0×3.0×4.0 mm^3^
datum diff	sub-501, sub-502, sub-503, sub-504 and sub-509 have float, instead of group std short
(some) oblique	
no slice timing	

**Group 5: anatomical**	
orient diff	sub-501, sub-502, sub-503, sub-504, sub-509 and sub-519 have RPI,
	instead of group std LPI
matrix size diff	much heterogeneity
oblique	

**Group 6: EPI**	
diff num of EPI	sub-601, sub-602, sub-603, sub-604, sub-605, sub-606, sub-607 and
	sub-620 only have 1, instead of group std 2
diff length of EPI	sub-601, sub-602, sub-603, sub-604, sub-605, sub-606, sub-607 and
	sub-620 have 240, 360, 480 or 724 time points, instead of group standard 130-133
oblique	
no slice timing	

**Group 6: anatomical**	
matrix size diff	sub-601, sub-602, sub-603, sub-604, sub-605, sub-606, sub-607,
	sub-612, sub-619, and sub-620 have 256×256×256, split with others having 256×256×176
oblique	

**Group 7: EPI**	
oblique	

**Group 7: anatomical**	
(some) oblique	

For each group, this displays cases of heterogeneity in basic dataset properties, as well as noteworthy values for checking or for informing processing choices. Items shown here might prompt verification with the source of the data collection, whether it has been downloaded from a shared repository or is being acquired locally.

Surprisingly, most groups (5 out of 7) contain heterogeneity of at least one basic dataset property. In Group 5, the EPI voxel dimensions of five subjects differ notably, which will affect SNR throughout the brain; additionally, the high anisotropy of the five outlier subjects can produce artifacts due to alignment and regridding. In Group 6, the numbers and lengths of runs vary within the group in complicated ways. These forms of heterogeneity can affect the statistical properties of estimated quantities, and lead one to question the appropriateness of combining these subjects in a group analysis (when not performing an explicitly large, multisite study, and these differences have a larger relative variance within the paradigm). Each of these items should lead to checking with the source of the data. If acquiring the data locally, performing the GTKYD check with each new subject can help identify problems or changes immediately, and minimize data waste.

Table 2 also contains absolute quantities that were notable either to prompt verification from the source of the data or to inform processing choices. As an example of the former, Group 2’s EPI values ranged from zero to over 2 × 10^6^; while FMRI datasets have no inherent units and this may not be a problem, these values are three orders of magnitude larger than typical dataset values, and therefore worth verifying their acquisition and reconstruction parameters to ensure that no numerical features (truncation, saturation, loss of contrast) have been introduced. Additionally, the EPI datasets in Groups 4, 5 and 6 did not contain slice timing information, which can be used for minor adjustment across the slicewise acquisitions. The lack of this information may be a reconstruction or distribution oversight, and hence obtainable. Finally, different software packages utilize obliquity information (the coordinate information that describes whether a dataset is acquired obliquely, away from simple cardinal orientations) differently during processing, such as: Applying it and regridding the data; ignoring it and effectively shifting coordinates; or leaving it in the header to be applied. Therefore, the choice and order of processing steps, particularly when it is present in an anatomical volume, may be affected by its presence. Here, we chose to remove obliquity of any anatomical volumes (while preserving the coordinate origin) as an initial processing step, to avoid issues with other software.

### APQUANT evaluation

The quantitative drop criteria listed in Table 1 were applied to the processed data, followed by APQUAL evaluations for each subject and, in several cases, GUI checks. A brief summary table of applying these stages of QC to the afni_proc.py-processed datasets is shown in Table 3, listing subjects in one of the three specified categories: Include (“high confidence to use in the hypothetical study”), Exclude (“high confidence to remove”) and Uncertain (“there is a question about whether to include”). The Supplementary Table 1 contains a table with more detailed descriptions for each subject.

**TABLE 3:** A brief summary of resting state FMRI dataset evaluations, based on the APQUANT, APQUAL and GUI QC checks.

QC evaluations (brief): Groups 1-7 (resting state FMRI)					
**Group 1 (I = 7, E = 8, U = 5)**			**508**	**E**	**APQUAL.vstat.artifact**
*sub*	*eval*	*comment*	**509**	**E**	**APQUAL.vorig.EPI**
**101**	**E**	**APQUANT.excl(‘flip guess’)**	510	I	
**102**	**U**	GUI.instacorr(odd patterns)	**511**	**E**	**APQUANT.excl(‘censor fraction’)**
103	I		**512**	**E**	**APQUANT.excl(‘censor fraction’)**
**104**	**E**	**APQUANT.excl(‘censor fraction’)**	**513**	**U**	APQUAL.vorig.EPI
105	I		514	I	
**106**	**E**	**APQUANT.excl(‘censor fraction’)**	515	I	
**107**	**U**	APQUAL.vorig.EPI(ringing feature)	516	I	
108	I		**517**	**U**	APQUAL.vorig.EPI
109	I		**518**	**E**	**APQUAL.vorig.EPI**
**110**	**U**	APQUAL.vstat.quality	**519**	**E**	**APQUAL.vorig.EPI**
**111**	**E**	**APQUANT.excl(‘GCOR’)**	520	I	APQUAL.regr.tsnr_final.quality
112	I				
113	I		**Group 6 (I = 10, E = 7, U = 3)**		
**114**	**E**	**APQUAL.vstat.artifact**	*sub*	*eval*	*comment*
**115**	**E**	**APQUANT.excl(‘flip guess’)**	**601**	**E**	**APQUANT.excl(‘GCOR’)**
**116**	**E**	**APQUAL.warn.flip**	602	I	
**117**	**U**	APQUAL.regr.TSNR_final-artifact	**603**	**E**	**APQUANT.excl(‘GCOR’)**
**118**	**E**	**APQUANT.excl(‘censor fraction’)**	604	I	
119	I		605	I	
**120**	**U**	APQUAL.regr.TSNR_final-artifact	**606**	**E**	**APQUAL.regr.corr_brain-quality**
			607	I	
**Group 2 (I = 0, E = 20, U = 0)**			608	I	
*sub*	*eval*	*comment*	**609**	**E**	**APQUANT.excl(‘GCOR’)**
2*	**E**	**GUI.instacorr(‘scanner artifact?’)**	**610**	**E**	**APQUANT.excl(‘GCOR’)**
			611	I	
**Group 3 (I = 9, E = 5, U = 2)**			**612**	**E**	**APQUANT.excl(‘GCOR’)**
*sub*	*eval*	*comment*	**613**	**E**	**APQUANT.excl(‘GCOR’)**
**301**	**U**	APQUAL.vstat.quality	614	I	
302	I		**615**	**U**	APQUAL.regr.corr_brain-quality
303	I		616	I	
304	I		617	I	
**305**	**U**	APQUAL.vstat.quality	**618**	**U**	APQUANT.warn(‘GCOR’)
306	I		**619**	**U**	APQUAL.vstat.quality
**307**	**E**	**APQUANT.excl(‘censor fraction’)**	620	I	
308	I				
**309**	**E**	**APQUANT.excl(‘censor fraction’)**	**Group 7 (I = 9, E = 10, U = 1)**		
310	I		*sub*	*eval*	*comment*
311	I		**701**	**E**	**APQUANT.excl(‘censor fraction’)**
312	I		702	I	
313	I		**703**	**E**	**APQUANT.excl(‘censor fraction’)**
**314**	**E**	**APQUANT.excl(‘censor fraction’)**	**704**	**U**	APQUAL.vstat.quality
315	E	APQUANT.excl(‘censor fraction’)	705	E	APQUANT.excl(‘censor fraction’)
316	E	APQUANT.excl(‘censor fraction’)	706	E	APQUANT.excl(‘censor fraction’)
			707	I	
**Group 4 (I = 0, E = 23, U = 0)**			708	E	APQUANT.excl(‘censor fraction’)
*sub*	*eval*	*comment*	709	I	
4*	E	GUI.instacorr(‘scanner artifact?’)	710	I	
			711	I	
**Group 5 (I = 7, E = 7, U = 6)**			712	E	APQUANT.excl(‘censor fraction’)
*sub*	*eval*	*comment*	713	E	APQUANT.excl(‘censor fraction’)
501	U	APQUAL.regr.TSNR_final-artifact	714	E	APQUANT.excl(‘censor fraction’)
502	U	APQUAL.regr.TSNR_final-artifact	715	E	APQUANT.excl(‘censor fraction’)
503	U	APQUAL.vstat.quality	716	E	APQUANT.excl(‘censor fraction’)
504	U	APQUAL.regr.TSNR_final-artifact	717	I	
505	I		718	I	
506	I		719	I	
507	E	APQUANT.excl(‘censor fraction’)	720	I	

The following abbreviations for evaluations (“eval”) are used: E, exclude; I, include; U, uncertain. Both Groups 2 and 4 were found to have artifacts in each of their datasets, and hence all categorized for exclusion. A more detailed summary is provided in the Supplementary Table 1, with further comments about most subjects.

In these tables, the QC comments are named hierarchically, in the following format: STAGE.type[.subtype](detail), using the terms listed in the previous section. For example, APQUANT.excl.(“flip guess”) represents the label for the left-right flip check within the exclusion criterion check during the APQUANT stage. Some “detail” elements are not contained within the brief table, but are included in the more complete Supplementary Table 1. This notation has been introduced to provide a clear, brief reference to the source of the particular QC criterion.

There were 139 total resting state subjects processed. As discussed further below, Groups 2 (20 subjects) and 4 (23 subjects) were found to have artifacts across all subjects, following APQUAL and GUI QC checks. Of the remaining 96 subjects, 42 were categorized to include in further analysis, 37 to exclude, and 17 were listed as uncertain. Of the 37 to exclude, 31 were evaluated as such using APQUANT criteria: 21 by censor fraction, 8 by GCOR, and 2 by left-right flip checking (though one additional subject was categorized as “uncertain,” primarily due to left-right flip checking, as discussed in the APQUAL section below). The left-right flip evaluations were always visually verified during the APQUAL stage. The quantitative GCOR value typically correlates highly with the APQUAL’s “regr.corr_brain” evaluation, as well.

The warning-level APQUANT criteria were additionally noted in subject evaluations (see the detailed Supplementary Table 1). In particular, these were combined with APQUAL criteria for determining additional “exclusion” or other categorizations, as described below.

### APQUAL evaluation

**FIGURE 2:**
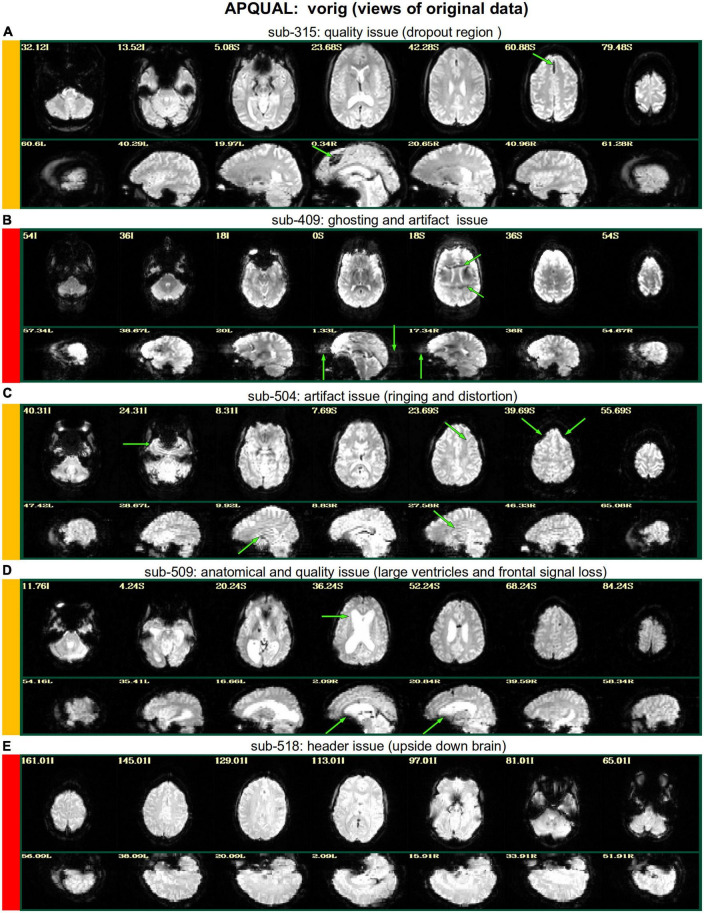
APQUAL examples for the “vorig” QC block: Visualizations of the original datasets (here, just the EPIs). In this figure and below, the colored bands to the left of each item denote whether the given QC item would suggest that the subject should be excluded (red), included (green) or leads to an “uncertain” evaluation (yellow); also, see Table 3 for brief, overall evaluations for each subject, and the Supplementary Table 1 for detailed QC comments. **(A)** The EPI contains a moderately sized dropout region (but it is mostly contained within the central sulcus). **(B)** This EPI contains severe ghosting artifact. **(C)** The inferior slices show a ringing artifact, and the frontal region is geometrically distorted. **(D)** This subject’s large ventricle may negatively affect alignment to template space, and there is notable dropout in the orbitofrontal region and subcortex. **(E)** The EPI is upside down, a significant header or data conversion problem.

**FIGURE 3:**
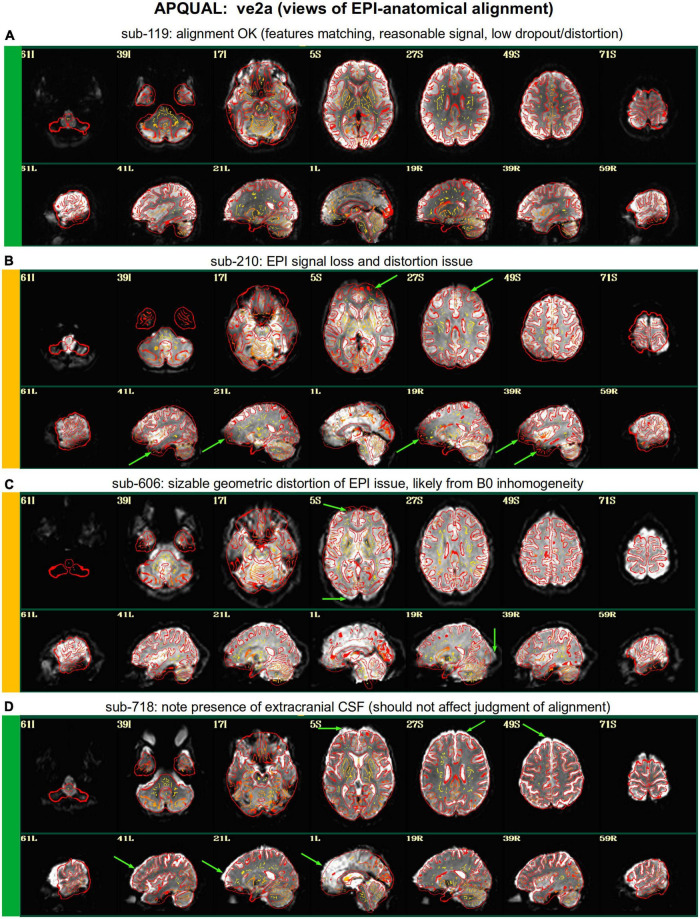
APQUAL examples for the “ve2a” QC block: Visualizations of the EPI-to-anatomical alignment (underlay = EPI; overlay = anatomical edges). **(A)** Structures appear generally well-registered. **(B)** There is notable EPI signal loss in the frontal and subcortical regions. **(C)** The EPI contains large distortions: Signal pileup in the anterior, and geometric stretching and signal attenuation in the visual cortex. **(D)** In judging EPI-anatomical alignment, interior structures matter most and CSF (bright, and highlighted with arrows) should be ignored.

**FIGURE 4:**
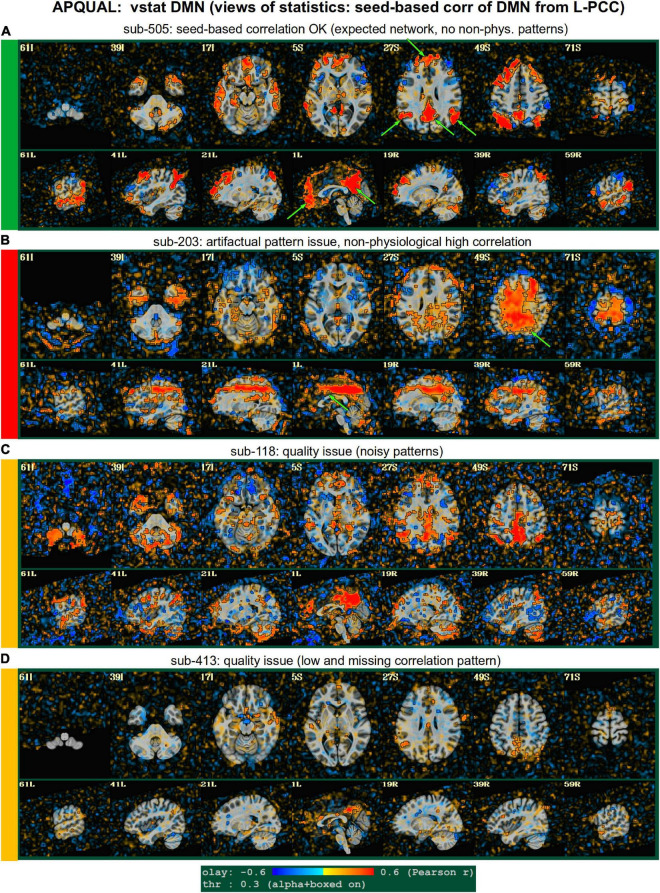
APQUAL examples for the “vstat” QC block: Visualizations of statistical information after regression modeling [here, the seed-based correlation map showing the default mode network (DMN]). The Pearson correlation values are overlaid, and are also used for thresholding, which is applied transparently: Suprathreshold voxels are opaque and outlined in black, while sub-threshold values are also shown but with opacity decreasing with value. This “highlighting” form of thresholding is applied here and below. **(A)** Expected regions are present, and high correlation regions show network-related spatial specificity (some noise, blurring, and asymmetry are expected). **(B)** A large, non-physiological region of high correlation is shown, and appears to be artifactual. **(C)** The expected network is discernible, but there is notable noise and distortion. **(D)** Almost no intra-network correlation is observed.

**FIGURE 5:**
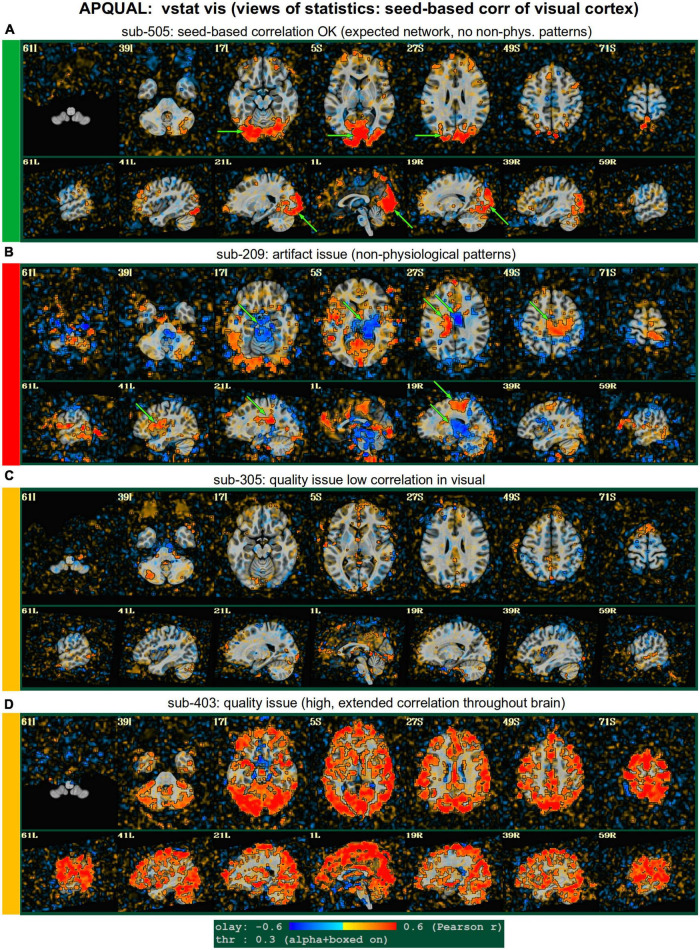
APQUAL examples for the “vstat” QC block: Visualizations of the statistical information after regression modeling (here, the seed-based correlation map showing the visual network). **(A)** Expected regions are present, and high correlation regions show network-related spatial specificity (some noise, blurring, and asymmetry are expected). **(B)** A large, non-physiological region of high correlation is shown, expanding across multiple tissue boundaries, and appears to be artifactual. **(C)** Almost no intra-network correlation is observed. **(D)** The high correlation pattern extends far beyond the expected network (to nearly all GM).

**FIGURE 6:**
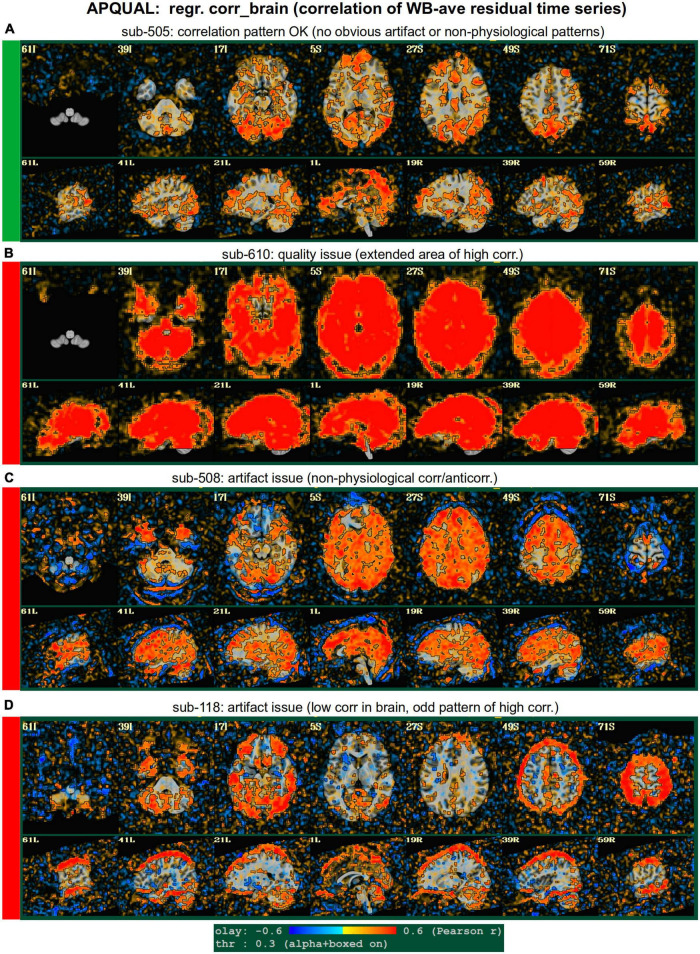
APQUAL examples for the “regr” QC block: Regression evaluation through the correlation pattern of the brain-averaged residual time series (“corr_brain” maps). **(A)** Regions of low-medium correlation are mainly located through the GM. **(B)** The whole brain volume correlates highly with the global average, suggestive of strong non-physiological signals remaining in the data. **(C)** High correlation extends through the intracranial regions, with large negative filaments, suggestive of strong non-physiological signals remaining in the data. **(D)** Strong patterns of high correlation remain in the data, outside of GM.

**FIGURE 7:**
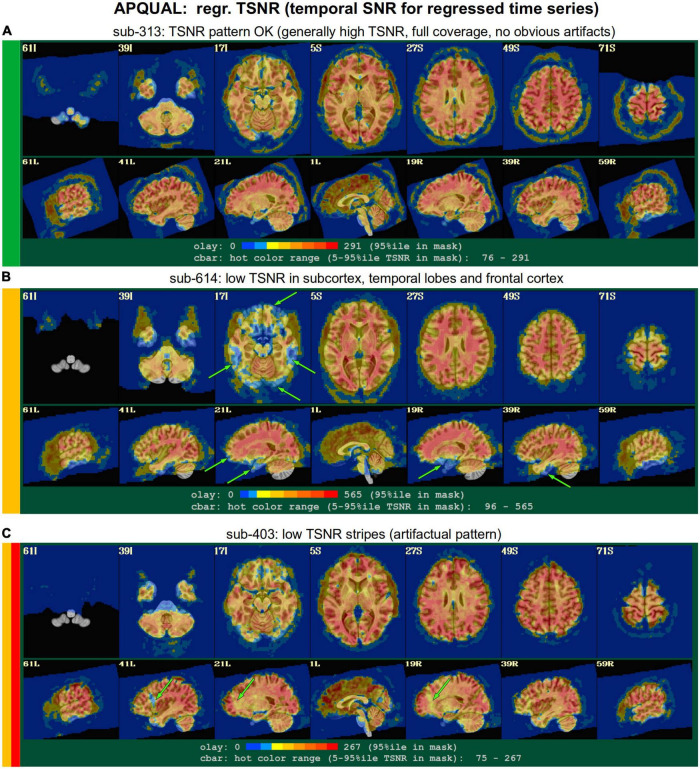
APQUAL examples for the “regr” QC block: TSNR maps of the final data after regression modeling (for each voxel, TSNR is the mean of the modeled time series divided by the standard deviation of the residuals). **(A)** TSNR is relatively constant and high throughout the brain volume (only very small regions of low signal, in the anterior temporal lobes). **(B)** Large regions of low-TSNR, particularly in the subcortex and orbitofrontal regions, which may impact cortical results. **(C)** Vertical strips of low TSNR are present, which may affect connectivity analyses (and which, after GUI-based investigation with InstaCorr, appear to be due to a significant artifact, shown in Figure 10, leading to subject exclusion; hence, the inclusion of red in the colorband to the left of the image).

**FIGURE 8:**
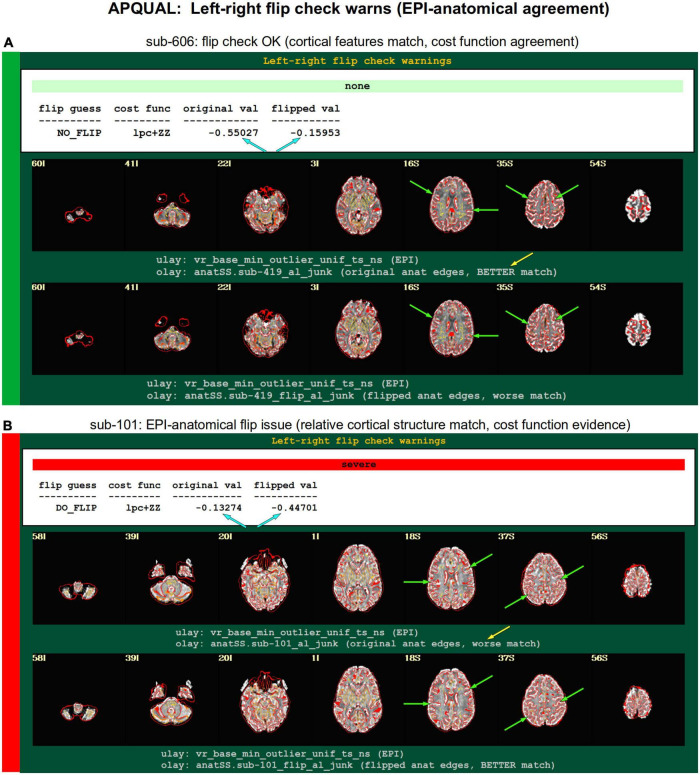
APQUAL examples for the “warns” QC block: Warnings created during processing, here for possible left-right flipping between the EPI and anatomical volumes. The warning field contains the APQUANT evaluation, based on cost function comparison (blue arrows), with its comment on the original (yellow arrow) and flipped EPI volumes. Importantly, images of each alignment result within the test are shown, for visual verification of the results. **(A)** The structures of the original EPI match well with the anatomical volume (and those of the flipped version do not), suggesting consistency. **(B)** The structures of the original EPI do not match well with the anatomical volume, while those of the flipped version do, suggesting inconsistency in the datasets.

**FIGURE 9:**
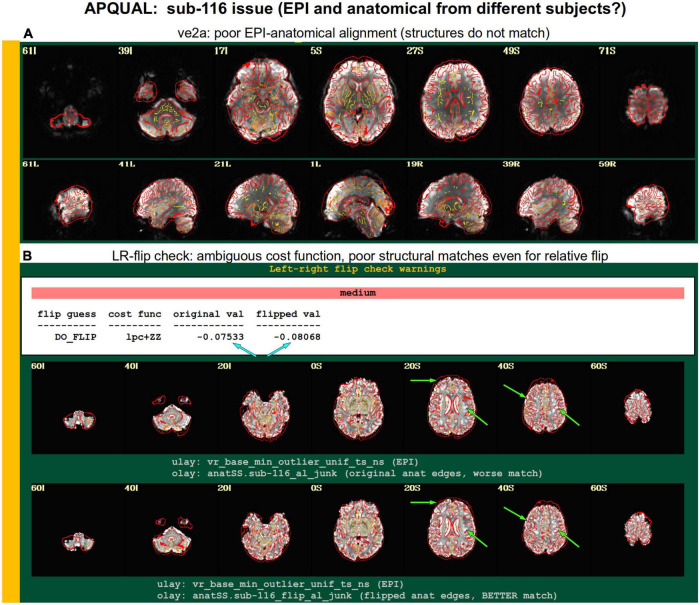
Combining APQUAL blocks: ve2a and warns (see Figures 3, 8). The structures of the aligned EPI do not match well with those of the anatomical, even though neither appears heavily distorted [ve2a, **(A)**]. The left-right flip check provides a “medium” level warning, because the cost function comparison is ambiguous [warns, **(B)**; see blue arrows]. Visually, neither the original nor flipped EPI matches well with the anatomical structures, even though all other subjects in the group had strong alignment. Since the structures appear to differ, this suggests that the EPI and anatomical volumes for this dataset may actually come from different subjects.

**FIGURE 10:**
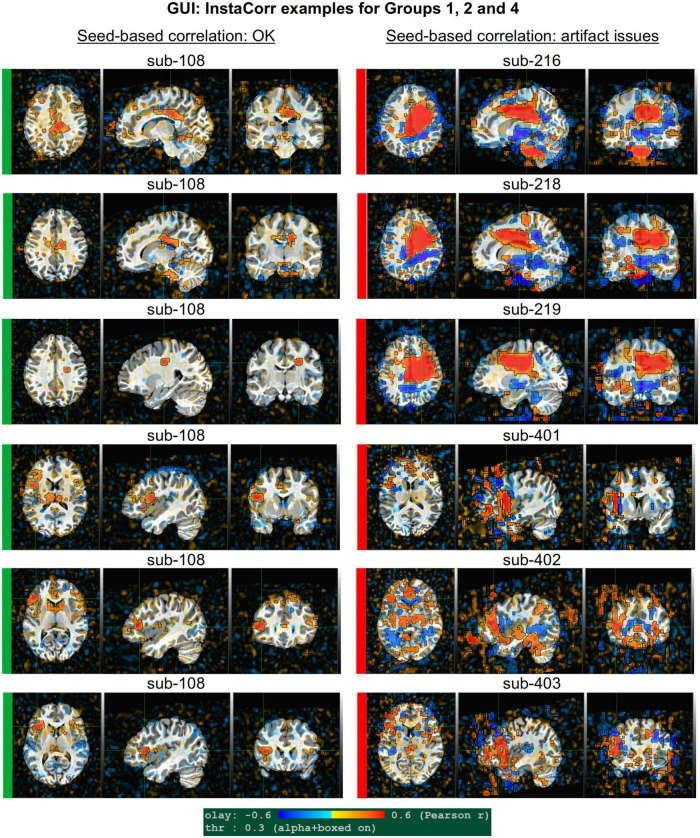
GUI examples of QC, using AFNI’s InstaCorr: This provides deeper understanding of the spatiotemporal patterns of the data through interactive driving of seed-based correlation. Several subjects in Groups 2 and 4 had difficult to interpret APQUAL QC results, particularly in seed-based correlation maps (vstat); upon further inspection here, it was apparent that those subjects contained large artifacts within the EPI datasets, as evinced by large correlation/anticorrelation patterns from seed locations in deep WM (Group 2) and extensive, non-physiological correlation/anticorrelation patterns from frontal GM/WM seeds (Group 4). In the end, these artifacts appeared to be present in all subjects of these groups, so that all were categorized for exclusion.

Figures 2–10 contain example images of the APQUAL evaluations, which are (by definition) qualitative and visual. Each figure shows multiple examples of the same APQC block from the HTML report. Each QC image is labeled with a colorband along its side, based on whether it would lead to excluding the subject (red), including the subject (green) or uncertain evaluation (yellow). Many images also contain arrows highlighting features of note.

#### vorig

Figure 2 shows QC examples from looking at one volume of the original EPI data (here, the “minimum outlier” volume from the EPI time series, which had the fewest outliers within the brain mask and was used as a reference for motion correction and alignment to the anatomical). In panel A, sub-315’s EPI shows a medium-sized patch of signal dropout. The associated anatomical volume contained a smaller spot at that location, so it is likely due to some local object (rather than a scanner artifact). This places a question of the full signal effects in this region, but since it is only moderate size and relatively constrained to the central sulcus, it might be reasonable to include the subject.

In Figure 2B, there is a strong ghosting signal present, as further investigated using InstaCorr. It is particularly noticeable throughout the central region of the brain, and, therefore, the signal patterns would be highly non-physiological, and the subject should be excluded. The subject in panel C has a smaller amount of ghosting and a “ringing” artifact in the inferior slices. The exact degree of signal effect is uncertain, hence the QC rating. In panel D, we see that sub-509 has extremely large ventricles, which reduce the quality of anatomical-to-template alignment, and may also reduce the quality of EPI signal. The subject also has a large amount of frontal and subcortical signal dropout, which renders inclusion uncertain.

Finally, there were multiple subjects in Group 5 who had upside-down EPI volumes, as shown in Figure 2E. Such large header errors warrant rejection, because the correct left-right designation is not possible to reliably ascertain *a posteriori*, without a marker. While it would be possible to try to fix the header and then assess results against the subject’s anatomical using AFNI’s left-right flip check, given the nature of this header issue one might not be sure of the correctness of the anatomical volume’s reconstruction. Therefore, given the high uncertainty of basic properties, such subjects should likely be excluded (though, in a different setting, one might contact the source of the data and query whether the initial reconstruction could be corrected).

#### ve2a

Figure 3 shows the alignment of an EPI volume (underlay) to the same subject’s anatomical (overlay, as edges). While EPIs typically contain geometric distortions (e.g., EPI distortion along the phase encode axis), affine registration is typically adequate to align most major structures to the higher-resolution and -detailed anatomical, as shown in panel A. However, EPI images often contain signal dropout, particularly bordering the sinus cavities, bordering the orbitofrontal cortex and subcortex. The ve2a block (views of EPI-anatomical alignment) provide useful images for assessing locations of dropout (as do TSNR maps, described below). Panel B shows several locations of poor signal strength and attenuation, which renders the suitability of sub-210’s data uncertain. Panel C shows a case where the geometric distortions make global EPI-anatomical alignment difficult (see the signal pileup in the anterior and attenuation/extension in the visual cortex).

An important point for judging EPI-anatomical alignment is exemplified in Figure 3D. The most important features to verify as matching are the tissue boundaries, sulci and gyri: The internal structures. At the edge of the brain, cerebrospinal fluid (CSF) can variously appear brightly, and make alignment details difficult to assess or create an impression of poor alignment. The CSF is particularly bright in Panel D (and for many subjects in Group 7), but the structural alignment still appears to be quite high (albeit in the presence of some geometrical distortions).

#### vstat.DMN

Figure 4 shows part of the “vstat” QC block, which provides views of statistics based on the regression modeling. For resting state FMRI, this includes seed-based correlation maps when the final data is in a recognized template space, and the images in this panel use a seed in the left posterior cingulate cortex [L-PCC; coordinate (5L, 49P, 40S) in the MNI template space], which is a standard part of the standard default mode network (DMN) along with medial prefrontal cortex and left/right inferior parietal lobules. This (and the other vstat seed-based vstat maps) provides a useful QC check for noise, artifact and modeling, since generally consistent spatial network patterns appear across age groups, species and alertness/sleep levels. Panel A shows what would be a typically reasonable result for a single subject map for sub-505: The higher correlation regions approximately follow the expected DMN pattern with acceptable specificity and approximate symmetry. Given the generally low SNR of FMRI, as well as length of scanning, one expects small noise patterns of correlation/anticorrelation. Note that here “transparent thresholding” is applied to the overlay, so that results below Pearson *|r|* = 0.3 are still observed, and brain masking is not applied: These features reduce the sensitivity of results to threshold value and allow for subtle patterns anywhere within the acquired FOV to be observed, which is vital for artifact detection (Taylor et al., 2022).

Figure 4B shows an example of an obvious artifact appearing in the correlation map of sub-203. The slice-wise nature of strong correlation throughout the brain is highly non-physiological, and strongly suggests this subject should be excluded from further analyses. Motion levels and other quantitative QC properties for this subject were not even at a warning level. The other two seed-based maps of the visual and auditory networks did not show obvious artifactual patterns, but the “corr_brain” map and “radcor” maps in the QC did show further extent of odd patterns. As described below, we also applied the GUI to investigate this subject (and others within Group 2), further verifying the presence of artifact (which unfortunately led to the exclusion of all subjects within Group 2).

Panels C and D of Figure 4 show other issues that can be arise in seed-based correlation maps: Noisiness (without an obvious artifact), which includes relatively high correlation/anticorrelation scattered around the FOV and/or mildly distorted patterns, as for sub-118; and widespread low or missing correlation patterns, as for sub-413 (and alignment quality was verified, so seed location did not appear to be obviously erroneous). In either case, the lack of strong artifact pattern makes it difficult to decide to exclude either subject from these images alone, and further investigations would be needed to avoid biasing the final group selection. (In these cases, the APQUANT stage showed suprathreshold censoring levels of 61% for sub-118, and the GUI-based InstaCorr check revealed notable artifact patterns in the frontal region for sub-413; therefore, from those separate criteria, each subject was excluded).

#### vstat.vis

Figure 5 shows another vstat visualization, for the visual network [seed located at coordinate (4R, 91A, 3I) in the MNI template space]. Panel A shows an expected correlation map for the same sub-505, which essentially contains high correlation in the V1/V2, V3, occipital areas and visual-associated areas. In contrast, panel B shows the presence of large patches of strong correlation and anticorrelation in other parts of the brain for sub-209. Furthermore, these patterns are not constrained by physiological or tissue boundaries. In total, this leads to excluding this subject (as noted above, GUI follow-up across Group 2 further verified extended artifacts).

In Figure 5C, a low/missing correlation pattern is observed for sub-305, again leading to an uncertain evaluation from this image. For this subject, the same low correlation was observed across all seed-based maps, but there was no obvious criterion for exclusion, and therefore the “uncertain” rating remained. In panel D, sub-403’s network map shows unexpectedly extensive regions of high correlation, throughout most of the gray matter (GM). While this “overfull” region of high correlation differs notably from the visual network regions, the lack of distinct, non-physiological patterning makes it difficult to exclude a subject from this image. (This subject’s APQUANT criteria were all below threshold, but as noted above, a GUI QC check with InstaCorr revealed that all subjects in Group 4 had a notable artifact within their dataset, leading to their exclusion).

#### regr.corr_brain

Figure 6 displays another volumetric visualization, which is the “regr” block’s “corr_brain” map: The brainwide average of the regression model’s residual time series (the “global signal”) is correlated with each voxel in the FOV. This essentially provides a visual assessment and corollary to the GCOR parameter (Saad et al., 2013), which is used as a warning and exclusion criterion in the APQUANT QC. Panel A shows a correlation map for sub-505, whose data had generally reasonable correlation maps (and a very subthreshold GCOR = 0.05). Much of the GM shows a generally positive and “medium-level” correlation, with typically low correlation in other tissues. This can be contrasted with sub-610, whose map has universally quite high correlation and leads them to being excluded (as did the associated GCOR = 0.47, in the APQUANT stage).

Figure 6C shows another problematic corr_brain map. While the GCOR = 0.08 for sub-508 is well below threshold, the relatively high correlation patterns across all tissues and anticorrelation boundaries appear to be artifactual. We note that this subject also displayed artifactual patterns in the vstat seed-based correlation maps. The high correlation patterns for sub-118 in panel D do not show the same whole brain coverage, but they do appear to be strongly non-physiological, and lead to this subject also being excluded. (Recall this subject’s “uncertain” noisy correlation map in Figure 3C, as well as the fact that censoring levels were also at a level for exclusion).

#### regr.TSNR

In Figure 7, TSNR maps for the final, regressed data are shown^3^. As typical TSNR ranges can vary with scanner site, the colorbar is defined relative to a 5–95% ile interval within the brain mask (providing the min-max values of the hot colors, respectively). Panel A shows a relatively good TSNR pattern: While there is some dropout in the orbitofrontal regions and temporal lobes for sub-313, such effects are present in nearly all FMRI and the TSNR strength is relatively constant across the brain and GM. If the low TSNR is not in a focal region of the study, then this subject would be fine to include in the subsequent analyses; for studies that include these regions of typical signal loss, one would have to adjust acquisition parameters to avoid problematic distortions. (Note that one can observe the tight FOV for this subject’s EPI, which would preferably be larger to avoid TSNR issues in the superior slices, as well).

The TSNR map for sub-614 in Figure 7B shows a larger area of dropout in the inferior regions of the brain. As shown in the images, a larger fraction of the temporal lobe, subcortex and orbitofrontal regions have notably lower TSNR than the rest of the brain. As whole brain connectivity studies often include these regions, it is likely that such differences in FMRI signal could affect the final results, depending on the hypotheses and exact paradigm. Therefore, this subject may not be appropriate to include in the study, and is rated “uncertain” from these images.

Figure 7C shows a TSNR map for sub-403 with relatively full whole brain coverage of constant TSNR, even in the inferior and subcortical regions. However, there are notable vertical stripes of low-TSNR that appear in each hemisphere in the anterior regions (see the sagittal slices). Such non-physiological patterns suggest some kind of artifactual signal issue, such as significantly strong ghosting, which may affect large areas of interest. Therefore, these patterns may mean that this subject would be inappropriate to include in further analyses. However, we note that in a follow-up QC analysis using InstaCorr in the AFNI GUI, these striped locations showed extreme and non-physiological patterns of correlation/anticorrelation (described further below, and see Figure 10). These low TSNR stripes were observed across Group 4, and the GUI follow-up revealed the same artifact in all subjects, leading to the exclusion of this group. Thus, in this group the low-TSNR striping was a hallmark of an artifact that always led to excluding a subject, but it is possible that in other datasets, that might not be the case. At the least, such patterns warrant detailed follow-up, likely using the GUI.

#### warns.flip

The APQC HTML contains a “warns” section that is comprised of the results of various automatic checks that occur during afni_proc.py processing (see list, above). Each has an associated warning level of “none,” “mild,” “medium,” “severe,” or “uncertain.” Figure 8 shows the results of a particular warning that spans the APQUANT and APQUAL QC: Checking for left-right flips between the EPI and anatomical volumes. Panel A’s results suggest that sub-606 does not show an inconsistency: The cost function value of the original data set is much lower than the flipped version (blue arrows; and note that cost functions are minimized in the alignment process), and the images below allow one to visually verify that the cortical patterns of the original EPI are much more consistent with those of the anatomical volume. NB: The structures of the superior cortex tend to be much less left-right symmetric than the inferior regions and subcortex, and therefore provide more convincing evidence.

Figure 8B shows an example of the quantitative flip-check strongly suggesting that sub-101’s original EPI and anatomical volumes have a relative left-right flip. This result is visually verifiable in the associated images. Since the *absolute* left-right definition cannot be known (without external indication such as a vitamin E tablet in the FOV), this subject would be excluded from further analysis. The data for sub-115 in this group similarly appeared to have a left-right flip.

A particularly interesting case of left-right flip check results is shown in Figure 9. Here, sub-116’s ve2a check initially showed a relatively poor EPI-to-anatomical alignment. Additionally, the left-right flip check provides a “medium” level warning, because the cost function values when using the original or flipped EPI are extremely close; in such as case, the recommendation whether to flip or not is difficult to interpret, as it is effectively “within the error bars” of the alignment’s cost function estimation. Looking at all of the images, it appears as if the cortical structures of the EPI and anatomical volumes do not match well in either case. Given that the EPI distortion is not very large and that the EPI-anatomical alignment for all other subjects from the site displayed excellent structural correspondence, these QC results suggest that the two volumes in sub-116’s dataste did not actually come from the same subject. When using a publicly downloaded dataset, this is only a supposition and cannot be directly verified, and, therefore, we are uncertain about whether to include this “subject” in further analyses.

### GUI evaluation with InstaCorr

The APQUAL and APQUANT items listed above provide useful QC information: The quantitative and visual aspects provide complementary aspects for efficiently and systematically understanding many aspects of the data. For example, the EPI and anatomical left-right flip check can be quantitatively evaluated, but should always be visually verified. As shown for sub-116 in Figure 9, data visualizations are sometimes even necessary for interpreting quantitative findings appropriately. However, in some cases even the APQUAL visualizations did not contain enough information to confidently make a QC evaluation. Therefore, the GUI stage of QC was used in several cases, in particular using the “run” script provided by afni_proc.py to efficiently start the AFNI GUI with InstaCorr set up, to explore the spatiotemporal properties of the EPI data.

Figure 10 shows a set of representative GUI snapshots when applying InstaCorr. As noted above, some of the correlation patterns for subjects in Groups 2 and 4 were not as expected: Some contained large patches of correlation and anticorrelation; some contained faint (subthreshold) patterns that were difficult to interpret; some contained extremely low or missing spatial patterns. For all subjects in these groups, the GUI follow-up revealed strong artifactual patterns in seed-based correlations, and example of these are shown for a subset of each group and contrasted with what might be considered a reasonable pattern at the same location in subject that did not appear to have artifacts (sub-108).

The seed location for each of the Group 2 subjects (sub-216, sub-218 and sub-219) is located in deep white matter (WM), which should have minimal patterns of correlation. As in the left column, one might expect a small, local patch of correlation even in WM, due to data blurring, remaining motion artifacts, vascular-driven BOLD response in WM, and more. However, the large patterns of high correlation/anticorrelation for each Group 2 subject spans tissue boundaries non-physiologically. Since these patterns overlap variously with GM, they do not appear possible to separate typical resting state connectivity analyses, and therefore all of Group 2’s subjects were categorized for exclusion.

InstaCorr analysis for Group 4 (sub-401, sub-402, and sub-403) revealed a different location of artifact, as shown in the lower panels of Figure 10. With a seed located in either the left or right frontal GM or WM, again strong patterns of high correlation and anticorrelation appeared, in this case alternating and even extending outside the brain. Again, these patterns are in contrast with expected local and/or localized symmetric patterns of correlation, depending on the GM/WM content of the seed region. These artifactual patterns throughout the frontal cortex GM also imply that resting state connectivity analyses would be strongly affected by non-physiological features, and therefore all of Group 4’s subjects were also categorized for exclusion.

### Group summary QC notes

After performing the detailed single subject checks listed above, it can be useful to summarize features or trends that appear across the group. These may be helpful for judging the overall applicability of a data collection for a particular study question. Additionally, these may aid planning future studies, by either replicating important features or by avoiding non-ideal aspects, possibly adjusting acquisition parameters. In general, the following overall properties of each group are based on the visualization methods described in the APQUAL stage.

Group 1 had several subjects with relatively low visual cortex correlation in vstat.vis seed-based correlation maps, even though the other network correlation maps were more standardly represented. There were some light vertical striping patterns in the frontal brain regions of the TSNR plots, suggesting some mild ghosting effects. Finally, in the individual motion parameter plots, the dP (translation along A-P axis) tended to have a noticeably linear increase across time, which might be due to frequency drift (e.g., Foerster et al., 2005) or even from settling into a pillow; while not necessarily a problem, this is an example of a group-wide feature in the data that is worth understanding, particularly if acquiring one’s own data.

Subjects in Group 2 had relatively high corr_brain maps, and the TSNR dipped noticeably in the center of the brain. The radial correlation (radcor) patterns were noticeably high centrally, and this led to discovering the presence of a strong artifact across all subjects, using InstaCorr. If subject data were still being acquired, such an artifact might encourage close examination of all datasets coming from that particular scanner.

Groups 3 and 4 each had relatively tight FOV for the EPI acquisitions. These might negatively affect signal quality in some boundary regions.

Group 4’s EPI volumes had quite short time series (123 points). The TSNR plots showed a strong vertical striping pattern, which led to the discovery of a notable frontal artifact across all subjects, using InstaCorr. The motion plots revealed a steady dP translation over time (as well as some notable linear trends in other parameters).

In Group 5, the basic acquisition features of voxel dimension and matrix size were quite heterogeneous. Non-linear alignment of the highly anisotropic EPI voxels (1.87 mm × 1.87 mm × 4.0 mm) produced slight swirls in patterns, which is one reason that acquiring anisotropic voxels is not recommended for standard group analyses; it also creates a grid-based dependence for the acquired data (e.g., which brain regions are averaged together depends on the orientation of a subject’s head in the scanner), a property that should be avoided. There was also noticeable signal loss in the orbitofrontal and temporal lobes, as well as the subcortex, which may lead to the exclusion of most of these subjects in some whole brain studies, depending on the specific regions of interest.

Group 6 also had a large heterogeneity in basic acquisition parameters, particularly in terms of number of EPI runs and run lengths, as well as matrix sizes. There was notable geometric distortion in the EPIs, particularly along the phase encode axis, with both signal pileup and attenuation; due to the different patterns of distortion, the phase encode direction may have been inconsistent across the group. TSNR was high across much of the brain, but low in the orbitofrontal and temporal lobes. There were relatively high values of the corr_brain (the correlation of the average residual signal across the brain).

Group 7 had notably bright CSF in the frontal portions of the brain in the EPI, but this did not appear detrimental to alignment or analyses. This group seemed relatively prone to motion, with many subjects having unusually high censor fractions.

## Results for task-based data collection

### GTKYD summary

Similar to the analysis of resting state FMRI, GTKYD was the first stage of checking each group’s data, and no subject exclusions were made from this step. The summary of basic dataset properties for the single group of task-based FMRI (Group 0, 30 subjects) is shown in Table 4. One subject’s EPI had a different orientation from the rest of the subjects. While all EPI volumes were acquired obliquely, only a subset of anatomical volumes were acquired obliquely.

**TABLE 4:** Summary of the first stage of task-based FMRI QC: GTKYD (“getting to know your data”).

GTKYD: “Getting To Know Your Data” results (task-based FMRI)	
**Property**	**Description**
**Group 0: EPI**	
orient diff	sub-010 has RIA, from group std RPI
oblique	
**anatomical**	
(some) oblique	

This displays cases of heterogeneity in basic dataset properties, as well as noteworthy values for checking or for informing processing choices. Items shown here might prompt verification with the source of the data collection, whether it has been downloaded from a shared repository or is being acquired locally.

The table of GTKYD checks for the task-based FMRI group is shown in Table 4. Here, one subject’s EPI had a different orientation than the rest. While all EPI volumes were acquired obliquely, only some of the anatomical volumes had obliquity information; as with the resting state data, we chose to deoblique these anatomicals as an initial processing step. Finally, no slice timing information was present for these EPI volumes.

### APQUANT

Table 5 shows a brief summary applying APQUANT exclusion criteria (itemized in Table 1) and additional APQUAL and GUI checks to the task-based FMRI group. The same subject dataset categorizations (described above): Include, Exclude and Uncertain. The Supplementary Table 1 contains a table with more detailed descriptions for each subject.

**TABLE 5:** A brief summary of task-based FMRI dataset evaluations, based on the APQUANT, APQUAL and GUI QC checks.

QC evaluations (brief): Group 0 (task-based FMRI)					
**Group 0 (I = 15, E = 7, U = 8)**					
*sub*	*eval*	*comment*			
001	I		**016**	**U**	APQUAL.vstat.quality
002	I		**017**	**E**	APQUANT.excl (‘fraction TRs censored’)
003	I		018	I	
004	I		019	I	
**005**	**U**	APQUAL.vstat.quality	**020**	**U**	APQUAL.vstat.quality
006	I		**021**	**U**	APQUAL.vorig.EPI
007	I		**022**	**E**	APQUANT.excl (‘fraction TRs censored’)
008	I		**023**	**U**	APQUAL.vstat.quality
**009**	**E**	APQUANT.excl (‘fraction TRs censored’)	**024**	**E**	APQUANT.excl (‘fraction TRs censored’)
**010**	**U**	APQUAL.vstat.quality	**025**	**U**	APQUAL.vstat.quality
011	I		**026**	**E**	APQUANT.excl (‘fraction TRs censored’)
**012**	**E**	APQUANT.excl (‘fraction TRs censored’)	**027**	**E**	APQUANT.excl (‘fraction TRs censored’)
**013**	**U**	APQUAL.vstat.quality	028	I	
014	I		029	I	
015	I		030	I	

The following abbreviations for evaluations (“eval”) are used: E, exclude; I, include; U, uncertain. A more detailed summary is provided in the Supplementary Table 1, with further comments about most subjects.

The task-based FMRI data from 30 total subjects were processed. Following the QC checks, 15 were categorized to include for further analysis, 7 to exclude and 8 were listed as uncertain. Each excluded subject had at least one APQUANT criterion that resulted in that categorization (and typically multiple ones, as well as APQUAL items; see the detailed Supplementary Table 1). Most of the “uncertain” categorizations were due APQUAL examination, particularly to visualization of the statistical results, which are described in the next section.

### APQUAL evaluation

**FIGURE 11:**
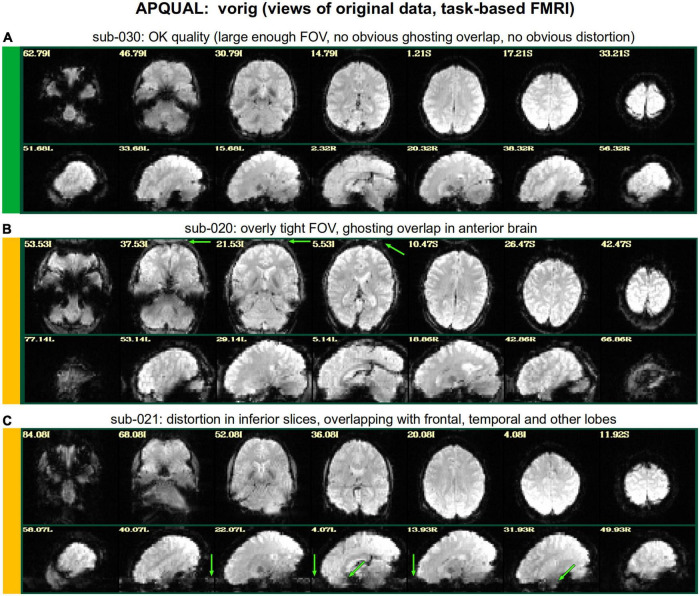
APQUAL examples for the task-based FMRI group from the “vorig” QC block: Visualizations of the original datasets (here, just the EPIs). See Table 5 for brief, overall evaluations for each subject, and the Supplementary Table 1 for detailed QC comments. **(A)** The EPI does not appear to have any major artifact, ghosting or distortion, and tissue contrast is reasonable. **(B)** The FOV of this volume is overly tight for this subject, so that there is ghosting of the posterior brain and skull which overlaps the anterior portion. **(C)** The inferior slices show a ghosting or phase distortion artifact—part of the frontal and temporal lobe regions are notably distorted.

**FIGURE 12:**
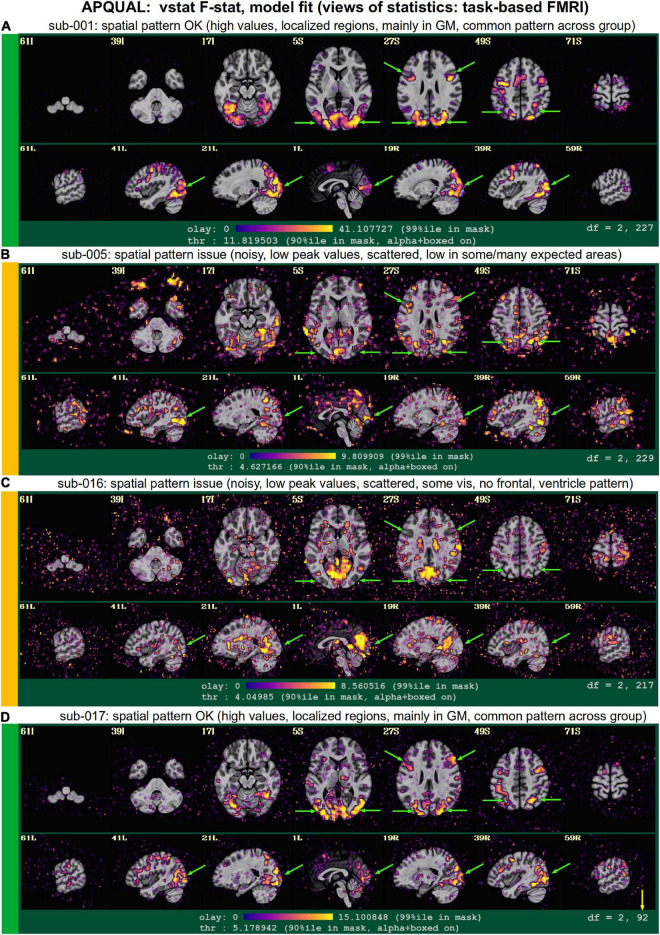
APQUAL examples for the task-based FMRI group from “vstat” QC block: Visualizations of statistical and modeling information after regression (here, the full F-stat from the regression modeling, highlighting regions of high model fitting). **(A)** High F-stat values are localized in GM (esp. visual cortex, and perhaps some in expected regions, if background knowledge is present), and this spatial pattern is fairly typical across the group (green arrows). **(B)** Compared to **(A)** the F-stat values are much lower (poorer fits) and less localized in GM, including the visual cortex, though the frontal regions in slice *Z* = 27S are observable; scattered noise has relatively high amplitude. **(C)** Compared to **(A)** F-stat values are much lower (poor fits) and less localized in GM, though part of the visual cortex is observed clearly; the ventricles have relatively high F-stat. **(D)** This dataset has similarly reasonable properties as dataset A, even though 57% of its time points were censored due to motion (note the second value in the degree of freedom count, df = 92, is much lower than the other volumes); this subject was still excluded, because of the automatic quantitative (APQUANT) criteria.

**FIGURE 13:**
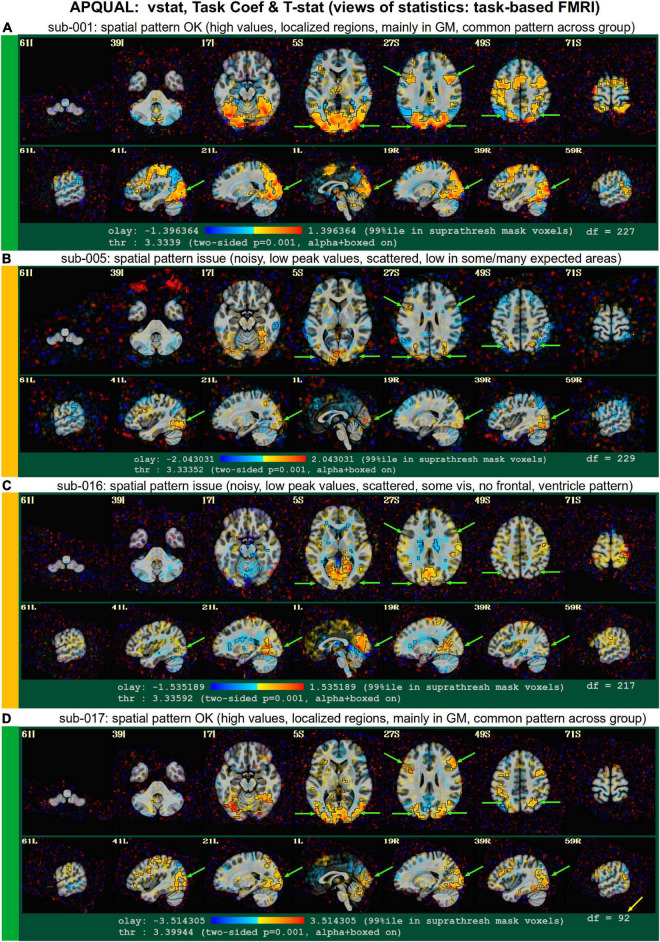
APQUAL examples for the task-based FMRI group from “vstat” QC block: Visualizations of statistical and modeling information after regression (here, the “TASK” stimulus coefficient is shown as the overlay colors, and its t-statistic values are used for thresholding). Each panel corresponds to that of Figure 12, though a different aspect of the modeling is shown here: Namely, the task stimulus coefficient that, after scaling, now has physical units of BOLD percent change, as well as the associated statistic (used for thresholding). Similar comments generally apply for each subject to those of Figure 12, but note that: For sub-005 [panel **(B)**], the high F-stat regions in frontal regions in *Z* = 27S were not strongly associated with this task, unlike in panels **(A,D)**; and for sub-016 [panel **(C)**], the ventricle pattern noted in the previous figure are negatively associated with the main task.

**FIGURE 14:**
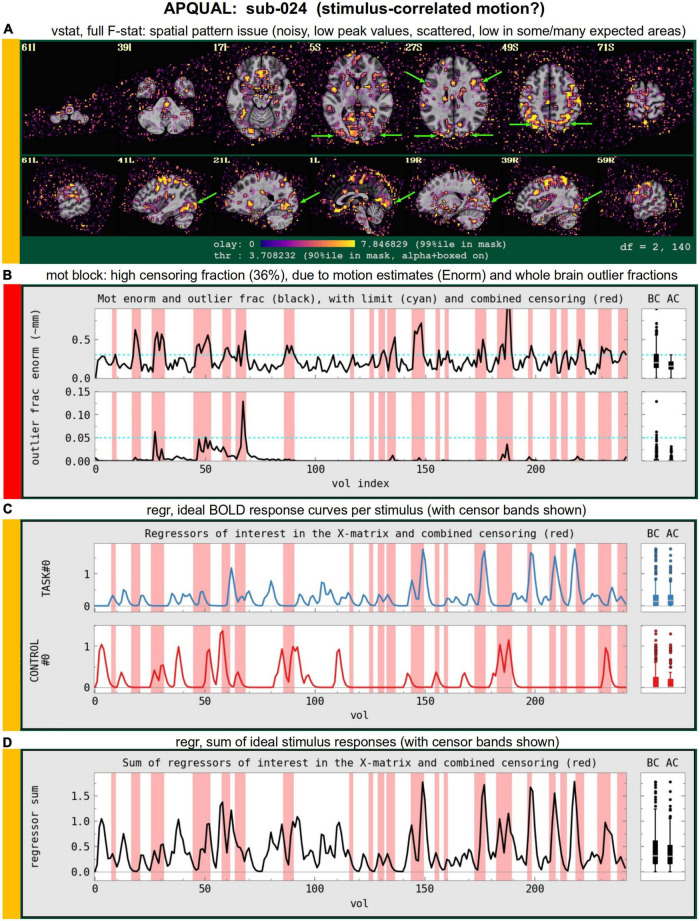
Combining APQUAL blocks: vstat (see Figure 12), mot (for combined motion estimates and censoring), and regr block plots of stimulus responses. The full F-stat map for sub-024 in panel **(A)** is noisy and shows relatively poor model fitting across the brain (cf Figures 12A, D). In trying to understand more about this subject’s data, the motion estimate responses are shown in panel **(B)**, where a large fraction of time points have been censored (>36%; shown in the red bands). Furthermore, in viewing the locations of censoring with respect to the ideal stimulus response curves for this subject [panels **(C,D)**], one sees that much of the motion appears to occur during many of the stimulus events. Thus, it is possible that this subject exhibits stimulus-correlated motion, which is particularly difficult to remove with modeling.

Figures 11–14 contain example images of the APQUAL evaluations for Group 0. These figures come from the APQC HTML report, of which most QC blocks are the same as for resting state FMRI. One exception is the vstat block, which shows F-stats and modeling coefficients (effect estimates) and associated statistics. The same colorband labels used for the resting state examples (see Figure 2) are used, as well as arrows to highlight features of note. In general, there were fewer QC issues with this group than for Groups 1–7. Therefore, we focus on different features in the overlapping blocks, as well as some of the stimulus-specific QC considerations.

#### vorig

Figure 11 shows QC examples from the “minimum outlier” EPI, used as a reference for motion correction and anatomical alignment. In panel A, sub-030’s volume does not display any obvious artifact or major distortion. The tissue contrast is also reasonable (some of the superior slices have slightly higher brightness, but the maximum value did not show saturation). In panel B, the FOV is much tighter for sub-020, and there is a notable ghosting artifact: The brain and skull from the posterior part of the brain is wrapped around to the anterior, and here appears to overlap with the brain volume. While different degrees of ghosting occur in many EPI acquisitions, there is a question here of whether the visible overlap suggests problematically strong signal interference in a non-negligible region of the study. In panel C, the inferior slices show the presence of ghosting or phase artifact. The distortion is limited to approximately the bottom ten slices, but this includes large portions of the frontal and temporal lobes (as well as other parts of the brain).

#### vstat

Figure 12 shows images of the full F-stat maps, which are part of each “vstat” block for task-based FMRI. As a ratio of explained variance to unexplained variance after regression, the full F-stat provides information on the relative model fit (higher values = better fit). These images provide a useful QC check for noise, stimulus modeling and motion reduction, though their details and expected patterns will (necessarily) vary strongly by paradigm. While there might be some expectation of regions of high F-stat that should be observed (e.g., the visual cortex when an on/off stimulus is presented visually; the motor cortex when button responses are used; some particular region from a previous study or theoretical rationale), it is difficult to apply an unexpected patterns as a drop criterion, unless an obvious artifact is observed, for example. Strong deviations across many subjects may be a sign of study design issues, subject unresponsiveness, stimulus timing issues or simply unexpected findings. While worth noting and commenting on, variations in statistical patterns will still be expected, and one must be careful not to bias results in the QC process.

In the present study, panel A of this figure (sub-001) shows what is likely a reasonable quality F-stat map for the present paradigm. A similar F-stat range (99% ile within the brain mask >40) and spatial pattern [high values in visual cortex, and left and right inferior frontal junction (IFJ); see green arrows in *Z* = 27S] were observed across many subjects, particularly among those with no obvious exclusion criterion. The high F-stat regions are localized in GM, and no obvious artifact or non-physiological patterns are observed.

Panels B and C show two subjects (sub-005 and sub-016, respectively) with generally lower F-stat values across the brain (99%ile within the brain mask <10). Note that motion and censoring levels for these subjects were not particularly, and no quantitative (APQUANT) criteria suggested excluding them. In the vstat images, the relative noise levels are higher and observed throughout the intranial region, and there are fewer obvious patterns of localized clusters of high F-stat. In B, relatively high F-stat clusters appear in the IFJ, but are barely observable in the visual cortex; in C, the opposite is the case, with the ventricles also showing surprisingly high F-stat. Such variations from the “standard” pattern are difficult to interpret, but are worrisome for including these subjects in group analysis. Further exploration was made using InstaCorr in the GUI (described below).

One of the additional vstat images automatically created was for the “TASK” stimulus, which is shown in Figure 13. This shows the coefficient (effect estimate) for the stimulus as the overlay, which here has units of BOLD% signal change, scaled to a 2 s stimulus, due to the inclusion of the scaling block and typical mean stimulus durations. Observing the coefficient (instead of just overlaying the statistic itself) is useful for interpreting the model results and judging their reasonableness. The stimulus (and contrast) plots also contain useful sign information, which is lacking in the F-stat images. Here, the locations of large effect and statistical significance typically mirror the high F-stat locations for panels A–D. Note that in panels B, the IFJ regions do not appear to have very strong “Task” stimulus response (relatively low magnitudes and statistics values). In panel C, the ventricles (which had high F-stat values in the same panel of Figure 13) show negative coefficients for this stimulus. While these images provide further useful details, again we note that the GUI was used to provide further information for sub-005 and sub-016.

#### vstat, mot, regr

Figure 14 shows several QC block results for sub-024. The vstat image in panel A shows a noisy statistical pattern and overall low peak F-stat values. Looking at other QC blocks or data aspects may provide useful information about why this dataset looks different, such as: Subject motion, lack of stimulus response, mismatched timing files, acquisition artifact or something else. This insight may be particularly important if checking datasets as they are acquired, to determine if study design or setup may be leading to a higher chance of having poor quality datasets.

For this figure’s sub-024, 36% of the time points were censored during processing (as well as >34% of each stimulus class’s response time), and the Enorm and outlier fraction plots (with threshold values and censoring bands) are shown in panel B. This high censor fraction led to this being categorized to be excluded in the APQUANT section, both because of the large information loss during stimulus events and due to the likely presence of remaining motion effects in the non-censored time points in practice; however, some subjects with high censor fractions do have stimulus response maps that appear to have reasonable quality (see panel D of Figures 12, 13), particularly if the motion is not strongly linked to stimulus events. Panels C and D show the ideal BOLD response curves for this subject, for both the individual stimuli and their sum, respectively, which also contain the censoring bands for reference. In this case, one might observe a possible trend of censoring during or immediately following stimulus events: It is possible that this subject has stimulus correlated motion, so that regression out motion regressors would also remove much of the stimulus-specific features. If several subjects contained such a correlation, then this would suggest the study design should be adjusted, or further procedures taken to reduce motion (e.g., giving specific instructions for the subjects, or having subjects practice the task and then provide feedback if motion appears high). Further QC investigations using an interactive GUI are described in the next section.

### GUI evaluation: InstaCorr

**FIGURE 15:**
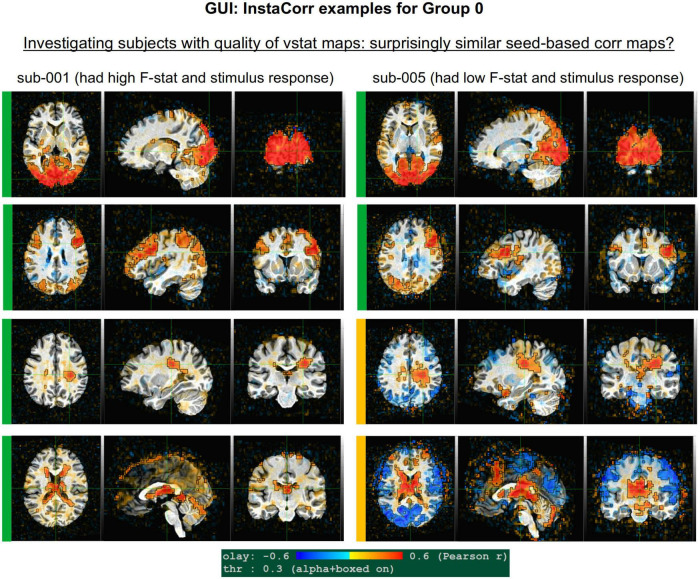
GUI examples of QC for task-based FMRI, using AFNI’s InstaCorr, to explore the spatiotemporal patterns of the EPI residuals with interactive seed-based correlation. The crosshairs show the seed location for two different subjects: sub-001 (left col) and sub-005 (right col); the same seed location is used, per row of images. The positive correlation responses are quite similar in both supra-threshold spatial coverage and magnitude for seeds in the visual cortex and IFJ (top and second rows, respectively). However, large, unexpected anticorrelation patterns in GM were observed for sub-005, leading to this subject being evaluated as “uncertain.”

Following the APQUANT and APQUAL stages described above, we further explored several of the datasets using the GUI, again using the “run” InstaCorr script provided by afni_proc.py. This can be useful generally to observe artifacts or systemic spatiotemporal features in the data. In particular, the APQUAL reports showed most subjects having strong task responses in visual areas, while others did not, some even when motion was low. This prompted a review using InstaCorr, which showed multiple features. Figure 15 shows InstaCorr images from sub-001 and sub-005 as respective examples of having strong task responses and not. While sub-005 had a poor task response, there were high correlations in the visual area (top row) and IJF (second row), akin to those of sub-001. Were we collecting this data locally, we would review the stimulus timing file creation, to be sure there were no mistakes. But sub-005 also shows unusual correlation and anti-correlation patterns between GM and deep WM, as well as with the ventricles. This led to the “uncertain” QC evaluation of sub-005.

### STIM evaluation

All subjects had essentially the same event onset timing, within 0.1 s, except for 2 subjects (sub-002, sub-026) for whom all events started 2 s later. Onsets (ignoring stimulus duration) were separated by times from 2.5 up to 18.5 s, with a mean of 7.5 s and a standard deviation of 3.5. When response time was applied for the duration, Control events had per-subject duration means from 0.51 up to 1.57 s, with an overall range of ≈0.0–2.43 s. Task events had per-subject means from 0.45 up to 2.65 s, with an overall range of≈0.0–4 s (with the latter being the maximum possible). ISI times (onset separations minus stimulus durations) ranged from 1.3 to 17.3 s, with a mean of 6.4 s. With well separated events, there were no concerning pairwise correlations between regressors. Though we note that since there were only two conditions, they were mildly predictive of each other, leading to typical pairwise correlations around −0.4 for those regressors of interest. The regression matrix condition numbers (computed as the ratio of the largest to smallest eigenvalues) very modestly ranged from 41.6 to 325.0, and were that high only due to correlations among the motion regressors.

### Group summary notes

The EPI volumes for Group 0 tended to have a tight FOV, particularly along the anterior-posterior brain axis. For several subjects, the strength of ghosting was large enough to be observed overlapping the frontal brain regions, which can create artifacts. There was notable EPI distortion in the inferior slices of several subjects, and the TSNR was generally low in the subcortex, temporal lobe and orbitofrontal lobe. Additionally, nearly every subject had the same timing onset; it is more common in newer studies that subjects would have randomized stimulus timing, though with similar statistical properties.

## Discussion

We have described a multi-stage process of QC for FMRI datasets. The stages are layered and complementary to help researchers understand their neuroimaging datasets, which themselves are complex and require many levels of processing that should be verified. We also introduced a standardized ontology to organize the recording and reporting of the QC procedure. These QC methods have grown and adapted over time, and will surely continue to do so, particularly through collaborations, encounters with more data, and neuroimaging community interactions such as the one at the core of this Research Topic project. It should be emphasized again that even beyond “including” and “excluding” subjects from a study, the larger–and perhaps more important—perspective of this process is to become confident of the contents of the data being analyzed. This principle applies to both public data that has been downloaded (which may or may not have been curated, or might have been curated with different analyses in mind) as well as to locally acquired data. Scanner upgrades, manual entry to scanner consoles, “automatic” console settings (that can change due to subject weight, for example), and more can affect the properties of acquired data in subtle but important ways. The researcher always has the responsibility to be aware of the dataset contents and their relative applicability for a given study.

Quality control, in the holistic sense emphasized throughout this paper, should start at the earliest stages of a study. Researchers should be “close to their data” from the very beginning, to reduce chances of downstream problems. Consider the following four steps:

1. Perform GTKYD, APQUANT and APQUAL checks, and review the results systematically.
2. Compare GTKYD, APQUANT and APQUAL results with previous studies.
3. (for task data) Review the duration and ISI statistics from any stimulus timing files.
4. Use the GUI to check steps of the processing (in particular running the automatically generated InstaCorr scripts) and look for any peculiarities.

When acquiring the first few subjects in a new project, it is important to perform a detailed review of the QC results across all stages, performing Steps 1–4; the same applies when starting with a shared data collection, examining a few subjects in detail. Any problems or questions should be dealt with immediately, to avoid data waste. After this in-depth review of the first few subjects, Steps 1–3 can be performed for the remaining subjects, with GUI investigations performed if any abnormalities are found.

The QC procedure of filtering subjects from further analysis is a subtle one: A researcher must balance the goal of basing results on reliable, non-artifactual data with the need to avoid introducing a bias. To date, there are no universal set of criteria for this process, and the heterogeneity of acquisition techniques, subject populations, research questions and analysis methods suggest this would be a challenging task. For any QC criterion, a desired trait is that in practice results are not overly sensitive to its thresholding value. For example, if a small change in a quantitative threshold leads to a large change in subjects excluded, one might try to find an alternate QC measure with a better delineation. One expects that over time and with more experience and feedback, QC measures will evolve to improve FMRI analysis.

Both quantitative and qualitative criteria have unique benefits; in many cases, they provide complementary checks and verifications. Quantitative ones are easier to apply uniformly, but in fact many quantities and their threshold values are based on much qualitative “training” and experience with datasets (and the many ways in which artifactual features can arise). Qualitative criteria require particular attention to be applied consistently, but, as evinced here, they provide a necessary perspective on data that is otherwise missed due to the inherently large data compression of derived quantities. If possible, qualitative criteria should not be central to the current analysis (to avoid bias), though that may not always entirely be possible (in which case, one must rely on the consistency of assumptions).

The primary QC criteria presented here relied on derived quantities (in the GTKYD, APQUANT, and STIM stages) and static images (in APQUAL) of the data. These are useful and able to be generated in automatic and systematic ways during processing (in the present work, *via* afni_proc.py). However, in some cases such items may only flag *potential* data quality issues, and a full understanding requires exploring the data itself more deeply. EPI datasets are inherently 4-dimensional, and occasionally too much information has been lost within the 1-dimensional scalar quantities or 3D image montages to understand an observed feature. Interactive exploration is then necessary to avoid “false rejections” of usable data (which is wasteful and may bias results) and “false inclusions” of problematic data (which introduce non-physiological features and again may bias or distort results). Here, we showed how GUI interactions could be used to more fully explore the underlying properties of the data, particularly with AFNI’s InstaCorr^4^.

In applying these QC principles and tools to the examination of this project’s eight publicly available datasets, we found quite a number of issues that ranged from incorrect header information (coordinate orientations, left-right flips) to ingrained data issues (temporally correlated artifacts, significant distortion, ghosting and dropout). In general, the exclusion criteria applied here were relatively light; some features such as inconsistent voxel size or acquisition parameters could be cause for rejection in an actual research study. Similarly, many datasets had distortion, dropout or other artifacts that particularly affected local brain regions, but the extent was judged as not severe enough for removal here. For a particular study’s hypotheses, though, such localized issues may render a subject’s data unusable. In the end, sizable fractions of these groups contained datasets that were categorized for exclusion, and another fraction with uncertain features for additional examination. Two groups contained systemic artifacts, likely rendering the data problematic for further analyses. This points to the necessity of performing full QC, and we hope that this Research Topic elevates QC’s role in the neuroimaging field: *Understanding* the data is an important part of processing it.

The data collections presented here provided an illustrative subset of the issues that exist in FMRI data. Many other problematic features can appear, such as major dropout from bad coils, zipper-striped artifacts, signal saturation, mechanical features in time series (e.g., from anesthesia devices), and more. Furthermore, different acquisition methods or processing choices will lead to different QC checks. For surface-based analysis, one would want to visualize the accuracy of the surface mesh estimation. For multi-echo FMRI, one might visualize maps of estimated T2*, as well as any temporal components projected out of the time series data. When combining data from several scanners, sites or even studies, the heterogeneity of datasets might prompt another layer of QC comparisons. It is important to note that QC criteria will never be set in stone, but will need to be adjusted based on the type of analysis, the subjects, and scanner and acquisition properties, which change over time.

The exact role of QC in determining final group outcomes is not well known (at present, at least). Certainly, cross-study accuracy, reproducibility and reliability should be improved by reducing artifacts in data collections. “Big data” does not preclude the need for reasonable QC—having a large fraction of problematic/artifact-heavy subjects can still be a problem whether the number of subjects is *N* = 50 or *N* = 5000. The QC process does require time and effort, but it is always a small fraction of the total effort that must be put into the study: Grant writing, pilot studies, subject recruitment, scanning, processing/reprocessing and (hopefully) publication. Choosing to save a relatively miniscule amount of QC time within a project can be quite costly, if the final results of a team’s work end up being based on unreliable data. Furthermore, if detailed QC is practiced at the early stages of data gathering, one would also expect it to greatly reduce the overall time of QC, because subtle issues could be observed and addressed before the number of subjects grows large.

There is often a desire to reduce all QC to a simplified, automated process. However, all quantities and thresholds used in QC procedures have been based on visualizing a large number of datasets and understanding their contents in depth. Even now, our current understanding of FMRI data quality is incomplete. Moreover, this process will always evolve: Study designs vary, and the technology of data acquisition is always changing. Image and time series visualization is the key to understanding data, and this layer should not be omitted from processing and quality evaluation. Ignoring visualization reduces the strength of QC, and hinders the ability to improve and develop new QC criteria—even quantitative ones. The QC results from this current project reinforce the importance of visualization: Researchers (particularly trainees just starting in the field) need to understand the data being processed, in order to avoid basing conclusions on unreliable datasets.

## Conclusion

This work addresses the question, “When should FMRI quality control be done?” with a resounding answer: “Early and often.” We present our approach to QC of FMRI data, organized as a set of stages that are integrated into standard processing with the AFNI software package. One aspect of this is evaluating subject datasets to be either included or excluded for a group level analysis. But the larger goal of the presented procedure is for researchers to deeply understand the contents of their data and to be sure of its appropriateness for their analyses of interest. This procedure applies when acquiring one’s own datasets, but remains vital when using publicly available or shared datasets. In all cases, a researcher has the responsibility to assess the properties of the data collection, and our approach here has been designed to facilitate this process with multiple layers of QC investigation. It includes a mix of scriptable, automated, visual and user-interactive checks that reinforce each other, many of which are created as standard outputs of the afni_proc.py pipeline generating tool. The stages begin with verifying the fundamental properties of the datasets, and continue through the single subject modeling. Using the real, public data provided in this Research Topic project, we have shown how each QC stage provided vital information about subjects for determining the suitability to include in further analyses. The range of issues present in this real data shows the continuing need for such QC procedures. We hope that researchers, data repository managers and particularly trainees in the field will find these methods and provided scripts useful when working with their own data.

## Data availability statement

Publicly available datasets were analyzed in this study. These data can be found here: https://osf.io/qaesm/wiki/home.

## Ethics statement

Ethical review and approval was not required for the study on human participants in accordance with the local legislation and institutional requirements. Written informed consent for participation was not required for this study in accordance with the national legislation and the institutional requirements.

## Author contributions

All authors listed have made a substantial, direct, and intellectual contribution to the work, and approved it for publication.

## Footnotes

1 See here for the main Project page: https://www.frontiersin.org/research-topics/33922/demonstrating-quality-control-qc-procedures-in-fmri and here for further details and download links for the datasets (https://doi.org/10.17605/OSF.IO/QAESM): https://osf.io/qaesm/wiki/home/.

2 By default, afni_proc.py creates images of the full F-stat and up to 4 additional coefficient + statistics pairs, depending on the number of stimuli and contrasts in the regression model. The user may specify any number of stimulus and contrast results to show, however.

3 TSNR can be variously defined in FMRI studies. Here, TSNR is the ratio of the mean of the voxel’s final time series to the standard deviation of its residual time series.

4 We note that the APQC HTML’s “vstat” block of images for resting state is essentially a quick, systematized version of InstaCorr exploration.
